# Contribution of short-term changes in a composite health score on pathways linking social disadvantage and health: evidence from the UK biobank prospective cohort study

**DOI:** 10.1016/j.eclinm.2026.104221

**Published:** 2026-09-29

**Authors:** Helene Colineaux, Rin Wada, Ruben Colindres Zuehlke, David Tang, Lola Neufcourt, Erlend Hoftun Farbu, Raphaële Castagné, Sameline Grimsgaard, Tom Wilsgaard, Torkjel M. Sandanger, Michelle Kelly-Irving, Cyrille Delpierre, Dragana Vuckovic, Marc Chadeau-Hyam

**Affiliations:** a Department of Epidemiology and Biostatistics, School of Public Health, Imperial College London, UK; b Equity Research Team, CERPOP, Université de Toulouse, Inserm, Université Paul Sabatier, Toulouse, France; c Department of Community Medicine, Faculty of Health Sciences, UiT the Arctic University of Norway, Tromsø, Norway

**Keywords:** Allostatic load, Embodiment, Social exposures, Cancer, Cardiovascular disease, Mortality

## Abstract

**Background:**

Chronic and repeated exposure to external stresses may be embodied and generate an adverse biological response, in-turn affecting health. We investigated the role of 4-year change in a Biological Health Score (BHS) in the pathway linking social exposures to all-cause mortality, atherosclerotic cardiovascular disease (AS-CVD), and cancer incidence.

**Methods:**

In this prospective cohort study, we analysed 20,048 participants (10,266 women and 9782 men, aged 37–73 years at baseline) from the UK Biobank prospective cohort with repeated biomarker assessments. The BHS was derived from 15 biomarkers covering cardiovascular, metabolic, inflammatory, liver, and renal systems. We examined associations between BHS change and six social exposures using sex-stratified linear models. Associations between BHS change and subsequent all-cause mortality, AS-CVD incidence, and cancer incidence (ascertained through registry linkage until December 2022) were estimated using sex-specific Cox proportional hazards models adjusted for baseline BHS and time between assessments, with sequential adjustment for additional exposures. Finally, we assessed whether BHS change mediated associations between social exposures and health outcomes using counterfactual mediation analysis.

**Findings:**

Total BHS increased over a mean interval of 4.3 years in both men and women, as did all sub-scores except the cardiovascular one. Lower education, low qualification, and financial difficulties were associated with greater increases in BHS. A 1-unit increase in BHS change was associated with: higher all-cause mortality in both men (hazard ratio [HR] 1.46, 95% CI 1.25–1.71) and women (1.26, 1.02–1.56); higher AS-CVD incidence in men (1.33, 1.13–1.57) and women (1.24, 1.03–1.48) and higher cancer incidence in men (1.16, 1.01–1.41) and women (1.29, 1.06–1.58). While no clear mediation by BHS change was observed for women, AS-CVD, or cancer, BHS change explained a small but statistically significant proportion of the association between low income and financial difficulties and mortality, in men.

**Interpretation:**

Whilst acknowledging the limitations of our dataset, we show that changes in a composite biological score over a few years, independent of age and baseline score, are socially-patterned and associated with subsequent mortality and cardiovascular disease, suggesting that repeated biological measurements may provide complementary information beyond single time-point assessments. Future research should extend these analyses to multiple repeated measurements, enabling the exploration of the dynamics of the embodiment of time-resolved external stresses and their effects on health.

**Funding:**

Research Council of Norway, European Commission.

**Research in context**

**Evidence before this study**

Social inequalities in health are well-documented, with disadvantaged groups showing higher morbidity and mortality. The concept of allostatic load provides a framework to capture how repeated or chronic stressors become biologically embodied, through cumulative solicitation of multiple physiological systems. Multisystem biological scores measuring this “wear-and-tear” predict morbidity and mortality and exhibit strong social gradients. However, most studies have relied on single time-point assessments and evidence on whether longitudinal changes in these scores may capture physiological dysregulation and mediate the impact of social disadvantage on disease incidence and mortality remains scarce.

**Added value of this study**

Using repeated biomarker measurements in the UK Biobank, we explored how a composite Biological Health Score (BHS) change over a 4-year period (between 2006–2010 and 2012–2013). We showed that increases in BHS were socially patterned, particularly by educational and economic disadvantage. Increases in the score predicted higher risk of cardiovascular disease, cancer and mortality (ascertained through registry linkage until December 2022), regardless of baseline values. In mediation analyses, BHS change accounted for a limited mediation proportion overall, but with statistically significant mediated proportions for economic disadvantage and mortality in men.

**Implications of all the available evidence**

Whilst acknowledging inherent limitations to the dataset and its generalisability, our results suggest that repeated multisystem biological measurements may capture complementary information beyond single time-point assessments and may provide useful indicators of evolving physiological dysregulation associated with social disadvantage. Such repeated measurements capture a holistic, multisystem, physiological state, associated with both socio-behavioural determinants and later health outcomes, and tend to add further information than measured at a single point in time. Further research using additional datasets, potentially including multiple repeated measurements, is warranted to validate and refine our findings.

## Introduction

Social inequalities have been consistently related to health disparities with social disadvantage being associated with poorer health outcomes and earlier mortality.^1^^,^^2^ Although substantial evidence links social experiences with behaviours and outcomes,^3^, ^4^, ^5^, ^6^ the mechanisms through which the social environment impacts health over the life-course remains to be better understood. The concept of social embodiment stipulates that the human environment, through physical, chemical, and psychosocial stresses triggers adaptive responses which are physiologically demanding. When intense or repeated, such exposures may over-solicit physiological systems ultimately leading to a physiological ‘wear-and-tear’ affecting the level of functioning of these regulatory systems.^7^^,^^8^ Multi-system scores, such as Allostatic load, has been proposed to capture these phenomena and have been associated with subsequent functional decline and mortality.^9^, ^10^, ^11^ These scores have been reported to exhibit strong social gradients^12^ and to reflect features of physiological dysregulation at different life stages.^13^ Expanding on the concept of allostatic load, we previously developed a Biological Health Score (BHS) that includes physiological systems not directly related to the stress response.^14^ The BHS was developed as an extension of traditional allostatic load scores, aiming to capture broader multisystem physiological dysregulations involved in biological embodiment processes and physiological wear-and-tear rather than stress-response systems alone. Our findings showed that individuals with lower educational attainment had higher BHS values, indicating higher biological risk, independently of other socially-patterned exposures and behaviours.^14^ This suggested that composite scores capture physiological patterns associated with social experiences and disadvantage. These scores have also been associated with subsequent all-cause and cause-specific mortality and cancer and cardiovascular diseases incidence,^15^ suggesting that multisystem physiological dysregulations may contribute to multiple chronic diseases through distinct pathogenic pathways.

Although the biological wear-and-tear measures are cumulative,^16^^,^^17^ the amplitude and the dynamics of these increments related to the occurrence of adverse life events and their effects on health has not been investigated. Most studies investigating multisystem biological dysregulation rely on single time-point assessments. The repeated assessment of UK Biobank provides a unique opportunity to investigate short-term changes in multisystem physiological dysregulation during mid-to-late adulthood. In the present study, using data from UK Biobank participants followed-up through registry linkage until December 2022, we investigate the role of the change in the BHS over 4 years on links between socially-patterned exposures and all-cause mortality, and the incidence of atherosclerotic cardiovascular disease (AS-CVD) and cancer. We first describe the evolution of key biomarkers and of the BHS over a 4 years period, and explore the association between changes in the BHS and (i) social factors, and (ii) prospective health outcomes. We finally explore, within a mediation framework, if and to what extent the links between social factors and health outcomes can be explained by the changes in the BHS.

## Methods

### Study design and participants

We used data from the UK Biobank prospective cohort studies, which includes 502,536 volunteers from the UK, aged 37–73 years at the time of enrolment between March 2006 and July 2010.^18^ Each participant provided informed consent and completed a computer-based questionnaire detailing life-course exposures, behavioural, sociodemographic factors along with medical history, and treatments. Clinical measurements were performed using standardised protocols at 22 assessment centres, and participants donated blood for long-term storage from which 30 biomarkers were measured^19^ at baseline and re-measured between December 2012 and June 2013. Prospective information on health was obtained through consented linkage to individual health records. UK Biobank data was accessed under application number 69328. Of the 502,536 participants, we included all those (N = 20,344) with two assessments and two measurements of biomarkers. We then excluded 14 lost participants (Left UK, withdrawn consent, etc.) and 282 participants with more than 50% missing data (Supplementary Figure S1) leaving 20,048 participants for subsequent analyses.

### Ethics

This study complies with the Declaration of Helsinki. Ethical approval was obtained from the National Research Ethics Service (Reference: 10/H0604/2). Participants gave written consent for blood sampling (McFall et al., 2014).

### Measures

#### Health outcomes

Mortality information was obtained through consented linkage to the national death registers and incident and prevalent health status by linkage to NHS central registers (cancer and hospital registers) until December 2022 and by nurse-administered questionnaires. Cancer cases included all-site malignant neoplasms (see Supplementary Table S1). Cardiovascular diseases were defined as atherosclerotic cardiovascular diseases (AS-CVD) including ischaemic heart diseases (angor, myocardial infarction...), diseases of arteries, arterioles and capillaries (including peripheral artery disease) and ischaemic stroke^20^ (Supplementary Table S1).

#### Biomarkers

A previously proposed,^15^ of the 30 biomarkers available in the UK Biobank dataset, we selected those related to the (i) Metabolic system (N = 4): Glycated haemoglobin (HbA1c), High-density lipoprotein cholesterol (HDL), Low-density lipoprotein cholesterol (LDL), Triglycerides (Tri); (ii) Cardiovascular system (N = 3): Systolic and diastolic blood pressure (SBP and DBP), pulse rate (PR); (iii) Inflammatory/immune system (N = 3): C-Reactive Protein (CRP), Insulin-like growth factor 1 (IGF-1) and Glycoprotein Acetyls (Glyc-A^21^); (iv) Liver function (N = 3): Alanine Transaminase (ALT), Aspartate Transaminase (AST), Gamma Glutamyl Transferase (GGT); and (v) Kidney function (N = 2): Creatinine (Cre) and Cysteine (Cyst).

#### Biological Health Score (BHS) calculation

Each of the (N = 15) pre-selected biomarker measurements were first log-transform and standardised. For protective biomarkers (IGF-1 and HDL), we multiplied the standardised biomarkers value by −1 so that an increase of any of the biomarker would correspond to a potential increased risk. For a given system and individual, the system-specific sub-score corresponds to the mean of the normalised values of biomarkers involved in the system, as proposed elsewhere.^22^ For the system S, the sub-score ${BHS}_{s,i,t}$ for individual i at time t is defined as.

$${BHS}_{s,i,t}=\sum_{k=1}^{N_{s}}\frac{x_{k,i,t}}{N_{s}},\text{where}$$

$x_{k,i,t}$ is the standardised measure of the k^th^ biomarker included in the system s measured for individual i at time t, and $N_{s}$ is the number of biomarkers in system S. The full BHS is defined as the mean of all system-specific sub-scores, thereby ensuring equal contribution of each physiological system regardless of the number of biomarkers included within each subsystem. Calculation were performed on the full datasets, as opposed to previous sex-specific analyses,^15^ to enable the exploration of sex/gender differences.^23^

#### BHS change calculation

We applied the same scoring methodology to the standardised differences between second and first measurement of each biomarker, i.e.:

$${BHS\Delta }_{s,i,t}=\sum_{k=1}^{N_{s}}\frac{d_{k,i,t}}{N_{s}},\text{where}$$

$d_{k,i}$ is the standardised measure of the differences between t=2 and t=1 of the non-standardised values of the k^th^ biomarker included in the system s measured for individual i. The overall BHS change score is therefore defined as the weighted mean of the normalised changes in all the biomarkers.

#### Socio-economic and psychosocial factors

In order to capture multiple dimensions of social resources,^24^ we examined several socio-economic and psychosocial variables: (1) age at completion of education and (2) qualification level (low/medium vs. high) as markers of cultural resources; (3) household income (below £18,000 vs. above) and (4) financial difficulties experienced in the last 2 years (yes or no) as markers of economic resources; (5) ability to confide in someone (rarely vs. yes/sometimes) and (6) loneliness (yes or no) as markers of socio-affective resources. Multi-categorical variables (qualification, income and ability to confide) were binarized for maintaining model parsimony, reproducibility, and statistical power. See detailed definition in Supplementary Table S2.

#### Covariates

We investigated a large set of available variables, collected at baseline from computer-based and nurse-administered questionnaires, as well as NHS central registers for some baseline health information. Definitions of prevalent health conditions incorporated available medication information when relevant, including self-reported medications coded according to the Anatomical Therapeutic Chemical (ATC) classification.^25^ After having excluded variables with more than 50% missing values (N = 82), 270 remaining variables were considered as potential determinants of BHS change and organised in the following eight domains (Supplementary Table S3): (i) History and family health (44 variables), (ii) Other socio-economic and psycho-social factors (19 variables), (iii) Home/Work/Urban environment (38 variables), (iv) Diet (30 variables), (v) Behaviours (44 variables), (vi) Anthropometrics (8 variables), (vii) Mental health at baseline (38 variables) and (viii) Physical Health at baseline (61 variables).

### Statistical analysis

For all variables, we performed a single stochastic imputation using MICE,^26^ applying restrictions on the predictor matrix to improve stability and using CART for sparse or collinear cases.

#### Selection of covariates and causal assumptions

To identify, among the 270 candidate covariates, those most likely to act as potential confounders or mediators for the purpose of adjustment in subsequent analyses, we selected variables jointly associated with changes in the BHS, independently of age, baseline BHS, time elapsed between measurements, and the six social factors of interest. Prior to analysis, all multi-category variables were hot-encoded, resulting in a total of 411 predictors. We then employed a stability selection approach using a series of LASSO regression models based on 100 random subsamples of the study population from which we estimated per-feature selection proportions as a proxy for feature importance. The hyperparameters of the model (the penalty controlling the sparsity of the model and the selection proportion threshold above which a feature is considered as stably selected, controlling the stability of the model) were calibrated using an in-house likelihood-based score.^27^ All models were adjusted for age, baseline BHS, time between measurements, and the six social factors of interest by ensuring that corresponding regression coefficients were not penalised. The selection procedure was stratified by sex and conducted for the total BHS score and for each sub-scores. Selected variables were subsequently classified as confounders or mediators according to *a-priori* assumptions specified in the Directed Acyclic Graph and time resolution (Fig. 1). Specifically, history-related variables were considered confounders of the associations between exposures and BHS change, whereas downstream variables (other social factors, environmental factors, behaviours, diet, anthropometrics, and health indicators) were classified as mediators. Since all exposures and covariates and were collected at baseline, this sequence was considered the most likely and common, but we cannot exclude reverse causality.

**Fig. 1:**
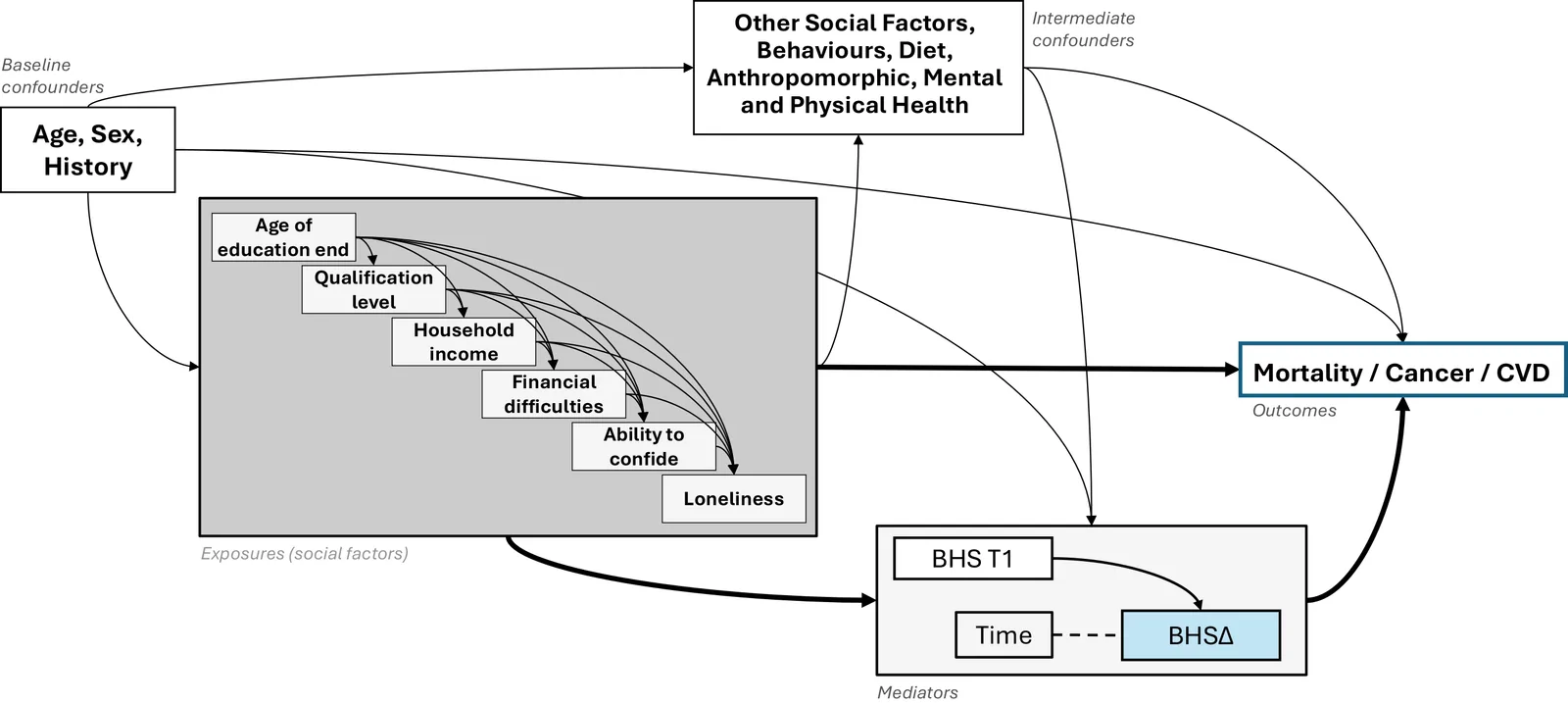
**Directed acyclic graph.** Directed acyclic graph describing the a priori causal and temporal hypothesis linking six social exposures of interest (age of education end, qualification level, household income, financial difficulties, ability to confide in someone, loneliness) to health outcomes (mortality, cancer incidence, atherosclerotic cardiovascular disease (AS-CVD) incidence) through the change of a biological health score (BHS) among participants of the UK Biobank study (United Kingdom), aged 37–73 years old. Age, sex and history were defined as baseline confounders and behaviours, diet, anthropomorphic measures, health at baseline as intermediate confounders.

#### Association between social factors and changes in the BHS

In an attempt to explore the total and direct effect of social factors on BHS changes, we modelled BHS change by each social factors using linear models, and sequentially adjusted (1) for age, baseline BHS and time between measurements, then (2) for previously-selected confounders and social factors conceptualised as upstream determinants (exploratory total effects) and finally (3) for previously-selected mediators and social factors conceptualised as downstream determinants (exploratory direct effects). A sensitivity analysis using original definitions of social variables has also been performed.

#### Association between changes in the BHS and health outcomes

Sex-specific multivariate Cox proportional hazard regression models, using age as timescale and adjusted for baseline BHS and time between 2 assessments, were fitted to estimate hazard ratios (HRs) for the BHS change (or system-specific sub-score) on (i) all-cause mortality and (ii) cancer incidence and (iii) AS-CDV incidence. In order to explore the factors by which the effect of BHS change on health may be confounded, we performed an attenuation analysis, whereby we sequentially adjusted our Cox models on factors previously found associated with BHS change and on social factors of interest, by time-ordered domains: history (M1), social and environmental factors (M2), behaviours (M3), diet (M4), anthropometric (M5), and health variables (M6). The proportional hazards assumption was assessed using scaled Schoenfeld residuals. For incident AS-CVD and cancer analyses, participants with prevalent disease at the second BHS assessment were excluded. Deaths occurring before the event of interest were treated as censoring events, and we therefore estimated cause-specific hazard ratios.

#### Mediation analysis

Mediation analyses were conducted within a counterfactual causal framework allowing for exposure-induced mediator–outcome confounding. The causal structure considered assumed that each social exposure could influence health outcomes both directly and indirectly through BHS change. Age, sex, history-related variables and upstream social variables, were considered baseline confounders, while downstream social factors, behaviours, diet, anthropometric measures, baseline health indicators, baseline BHS and time between two measurements, were treated as intermediate confounders, as these variables could be affected by the exposure while also influencing the mediator and the outcome.^28^

For each social exposure (age at end of education, qualification level, household income, financial difficulties, ability to confide in someone, and loneliness), mediation effects were estimated using the parametric g-formula implemented using Monte-Carlo simulation, as implemented in the CMAverse^29^ package. This approach relies on specifying parametric regression models for the mediator and the outcome, conditional on the exposure and confounders. The mediator model was a linear regression for the change in BHS, conditional on the exposure, baseline confounders, BHS, and intermediate confounders. The outcome model was a Cox proportional hazard model for each time-to-event outcome (all-cause mortality, AS-CVD, and cancer incidence), conditional on the exposure, mediator, baseline confounders, and intermediate confounders. A sensitivity analysis using alternative definitions (low vs. high/medium for qualification, below £52,000 vs. above for income and (rarely/sometimes vs. always for ability to confide) has also been performed.

Let Y denote the outcome, A the exposure, and M the mediator. Let a and $a^{*}$ denote the active and reference exposure levels, respectively, as defined in the Measure section. Counterfactual outcome expectations were estimated through a Monte-Carlo implementation of the parametric g-formula. First, the mediator model was used to predict the distribution of the mediator under specific exposure levels. For each individual, mediator values $M_{a}$ were randomly drawn from the predicted distribution of the mediator under exposure A=a, assuming normally distributed residuals from the fitted mediator model. These simulated mediator values were then inserted into the outcome Cox model to generate counterfactual predicted outcome hazards. This procedure was repeated for each individual and averaged across the population to estimate expected counterfactual outcomes under different intervention scenarios.

Using this framework, we estimated randomised analogue mediation effects, which decompose the total effect of the exposure without requiring independence assumptions. In these estimands, the mediator is not deterministically fixed but randomly drawn from the distribution that would be observed under a given exposure level. Specifically, the randomised analogue direct effect (rNDE) compares expected outcomes when the exposure is set to the active vs. reference level while the mediator is randomly drawn from the distribution observed under the reference exposure:

$$rNDE=\frac{E\left[Y_{aM_{a^{*}}}\right]}{E\left[Y_{a^{*}M_{a^{*}}}\right]}$$

The randomised analogue indirect effect (rNIE) compares expected outcomes when the exposure is fixed but the mediator is drawn from the distributions corresponding to the two exposure levels:

$$rNIE=\frac{E\left[Y_{aM_{a}}\right]}{E\left[Y_{aM_{a^{*}}}\right]}$$

The randomised total effect (rTE) on the ratio scale is obtained as the product of these two quantities:

rTE=rNDE×rNIE,

which corresponds to the contrast between the expected outcome when the exposure is set toaand the mediator is drawn from its distribution undera,and the expected outcome when the exposure is set to$a^{*}$and the mediator is drawn from its distribution under$a^{*}$.It can be interpreted as a marginal hazard ratio. The proportion mediated (PM) on the ratio scale was then derived as:

$$PM=\frac{rNDE\;\times \;\left(rNIE\;-\;1\right)}{TE\;-\;1}$$

Uncertainty was quantified using non-parametric bootstrap confidence intervals based on 1000 replicates. Mediated proportions were reported only when evidence for a non-null total effect of the social exposure on the outcome was observed.

All statistical analyses were performed using R v4.3.1 in the RStudio environment. All the codes, figures and tables are available upon request.

### Role of the funding source

The funders had no role in the design and conduct of the study; collection, management, analysis, and interpretation of the data; and preparation, review, or approval of this manuscript.

## Results

Of the 20,048 participants included in the analyses (10,266 women and 9782 men), the mean age at the first biomarker assessment was 57.1 years (standard deviation [SD] = 7.4). The mean time between the two biomarker assessments was 4.3 years (SD = 0.9). The mean age at completion of full-time education was 18.8 years (SD = 3.3), and 28.7% of participants had a low level of educational qualification (Table 1). Compared to men, women were more likely to have a low level of qualification (30.2% vs. 27.0%), to report a household income below £18,000 (18.4% vs. 15.7%), and to report loneliness (17.3% vs. 12.2%). However, women were less likely than men to report fair or poor self-rated health (17.0% vs. 20.1%). Relative to the broader UK Biobank cohort, our study population included a lower proportion of women (51.2% vs. 54.4%), older participants at baseline (mean age 57.1 vs. 56.5 years), less participants reporting financial difficulties (8.3% vs. 12.4%), and fair or poor overall health (18.5% vs. 25.7%).

**Table 1:** Characteristics of UK Biobank participants included in the repeat assessment study component (United Kingdom, baseline assessment 2006–2010 and repeat assessment approximately 4 years later).

	Women	Men	Total	All UKBB
Total N (%)	**10,266 (51%)**	**9782 (49%)**	**20,048**	**502,460**
Age at the first assessment (years)	56.57 (7.3)	57.72 (7.5)	57.13 (7.4)	56.53 (8.1)
Time between two assessments (years)	4.33 (0.9)	4.29 (0.9)	4.31 (0.9)	–
Social factors of interest				
Age of education end	18.74 (3.2)	18.88 (3.4)	18.81 (3.3)	17.39 (2.9)
Low qualification	3101 (30%)	2643 (27%)	5744 (29%)	132077 (33%)
Household income <18 K	1888 (18%)	1534 (16%)	3422 (17%)	97192 (23%)
Financial difficulties	831 (8%)	834 (9%)	1665 (8%)	61316 (12%)
Rarely able to confide	1559 (15%)	2348 (24%)	3907 (20%)	99073 (21%)
Feeling lonely	1771 (17%)	1196 (12%)	2967 (15%)	91393 (19%)
Behaviours, Diet and anthropometrics				
Total DASH score (0–5 pts)	3.40 (1.4)	3.03 (1.4)	3.22 (1.4)	3.10 (1.4)
Never smoked	6633 (65%)	5199 (53%)	11832 (59%)	273497 (55%)
>13 doses alcohol a week	2184 (21%)	4032 (41%)	6216 (31%)	112335 (32%)
Body Mass Index (kg m-2)	26.45 (4.9)	27.30 (4.0)	26.87 (4.5)	27.43 (4.8)
Waist Hip Ratio	0.81 (0.1)	0.93 (0.1)	0.86 (0.1)	0.87 (0.1)
Health				
General score: fair or poor	1740 (17%)	1968 (20%)	3708 (19%)	128129 (26%)
3 or more treatments	2258 (22%)	1884 (19%)	4142 (21%)	133760 (27%)
Prevalent diabetes	277 (3%)	530 (5%)	807 (4%)	26660 (5%)
Prevalent High Blood Pressure	2198 (21$)	2758 (28%)	4956 (25%)	138565 (28%)
Prevalent AS-CVD	371 (4%)	855 (9%)	1226 (6%)	36617 (7%)
Prevalent or previous cancer	1012 (10%)	657 (7%)	1669 (8%)	47227 (9%)

Characteristics of 20,048 UK Biobank participants (10,266 women and 9782 men) with two biological assessments available, after imputation of missing data. Participants were aged 40–73 years at baseline (mean age 57.1 years) and were followed over a mean interval of 4.3 years between assessments. Categorical variables are described by numbers and percentages, n (%) and continuous variables are described by means and standard deviations, m (sd). The p-value corresponds to a Wilcoxon test for continuous variables and a Pearson Chi-square test for categorical variables (given for the entire variable if there are several categories). The column “All UKBB” presents the corresponding characteristics for the full UK Biobank cohort (N = 502,460).

### Description of BHS at baseline and change over time

The total BHS score increased between two time points (+0.04, SD = 0.3), more strongly in women (+0.07, SD = 0.3) than in men (+0.01, SD = 0.3). Overall, all sub-scores, except the cardiovascular score, increased between two time points (Table 2). Direction of the change was consistent in both sexes for the cardiovascular (decreasing), inflammatory and renal (increasing) scores, while the metabolic and liver scores showed an increase in women and a decrease in men. We also see that change is consistently lower in those with higher baseline BHS at baseline (Supplementary Figure S2b). Across biomarkers, largest increases over the 4-year period were observed for cystatin C, creatinine, and HbA1c (+20%, +16%, and +14% of the SD, respectively), as well as systolic blood pressure among women (+14%) (Supplementary Table S4, Supplementary Figure S3). In contrast, HDL cholesterol, diastolic blood pressure (DBP), and pulse rate showed the largest decreases (−23%, −14%, and −14%, respectively). The decrease in DBP was more pronounced in men (−21%) than in women (−8%).

**Table 2:** Baseline biomarker levels and changes in biomarker levels, subsystem scores and Biological Health Score among UK Biobank participants with repeat assessments (United Kingdom, mean follow-up 4.3 years).

	Women		Men		Total	
	Baseline	Change	Baseline	Change	Baseline	Change
Cholesterol LDL (mmol/L)	3.62 (0.9)	0.07 (0.7)	3.47 (0.8)	−0.06 (0.8)	3.55 (0.9)	0.01 (0.7)
Cholesterol HDL (mmol/L)^a^	1.61 (0.4)	−0.10 (0.4)	1.29 (0.3)	−0.08 (0.3)	1.46 (0.4)	−0.09 (0.4)
Triglycerides (mmol/L)	1.52 (0.9)	0.11 (1.0)	1.91 (1.1)	−0.05 (1.1)	1.71 (1.0)	0.03 (1.1)
HBA1c (mmol/mol)	35.45 (5.2)	0.82 (3.8)	35.85 (6.1)	0.70 (4.1)	35.65 (5.7)	0.76 (4.0)
Metabolic system	**−0.14 (0.6)**	**0.05 (0.4)**	**0.15 (0.6)**	**−0.04 (0.5)**	**0.00 (0.6)**	**0.00 (0.4)**
Systolic Blood pressure (mmHg)	134.67 (18.7)	2.63 (15.0)	140.59 (16.9)	−0.11 (14.7)	137.56 (18.1)	1.29 (14.9)
Diastolic Blood pressure (mmHg)	79.73 (9.8)	−0.85 (8.1)	83.50 (9.6)	−2.03 (8.6)	81.57 (9.9)	−1.42 (8.3)
Pulse Rate (bpm)	68.92 (10.1)	−1.28 (8.7)	66.87 (11.3)	−1.66 (9.5)	67.92 (10.7)	−1.47 (9.1)
Cardiovascular system	**−0.08 (0.8)**	**−0.02 (0.6)**	**0.09 (0.7)**	**−0.13 (0.6)**	**0.00 (0.7)**	**−0.07 (0.6)**
CRP (mg/L)	2.46 (4.3)	−0.08 (4.6)	2.19 (4.1)	0.10 (5.1)	2.33 (4.2)	0.01 (4.9)
Glycoprotein Acetyls (mmol/L)	0.80 (0.1)	0.02 (0.1)	0.80 (0.1)	0.01 (0.1)	0.80 (0.1)	0.01 (0.1)
IGF-1∗ (nmol/L)	21.05 (5.7)	0.70 (3.9)	22.04 (5.3)	0.46 (3.6)	21.53 (5.5)	0.58 (3.8)
Inflammatory system	**0.04 (0.7)**	**0.10 (0.6)**	**−0.05 (0.7)**	**0.08 (0.6)**	**0.00 (0.7)**	**0.09 (0.6)**
AST (U/L)	24.50 (8.5)	0.88 (11.3)	27.81 (8.9)	0.04 (9.8)	26.11 (8.9)	0.47 (10.6)
ALT (U/L)	20.26 (12.3)	0.38 (13.2)	26.57 (13.6)	−1.12 (13.5)	23.34 (13.3)	−0.35 (13.4)
GGT (U/L)	29.04 (29.2)	0.58 (25.6)	41.72 (36.9)	−1.07 (30.8)	35.23 (33.8)	−0.22 (28.3)
Liver system	**−0.28 (0.8)**	**0.08 (0.7)**	**0.30 (0.8)**	**−0.05 (0.7)**	**0.00 (0.9)**	**0.02 (0.7)**
Creatinine (μmol/L)	63.94 (11.8)	2.05 (10.0)	81.08 (13.9)	2.95 (12.1)	72.30 (15.5)	2.49 (11.1)
Cystatin (mg/L)	0.86 (0.1)	0.03 (0.1)	0.92 (0.1)	0.03 (0.1)	0.89 (0.1)	0.03 (0.1)
Renal system	**−0.42 (0.8)**	**0.17 (0.5)**	**0.44 (0.7)**	**0.19 (0.5)**	**0.00 (0.9)**	**0.18 (0.5)**
**Total**	**−0.18 (0.4)**	**0.07 (0.3)**	**0.19 (0.4)**	**0.01 (0.3)**	**0.00 (0.5)**	**0.04 (0.3)**

Means and standard deviations are reported for 20,048 UK Biobank participants (10,266 women and 9782 men) who completed two biological assessments approximately 4 years apart. Baseline z-scores were standardised to have a mean of 0 and a standard deviation of 1 in the study population. Follow-up z-scores were calculated using the same baseline means and standard deviations, allowing changes to be interpreted as deviations from the baseline population distribution rather than relative changes within the follow-up sample.

a Distributions of biomarkers marked with a are considered protective, so the sign was changed for the score calculations.

### Association between social factors and BHS changes

The multivariate stability selection procedure identified 17 variables to be included as adjustment variables in women (birth weight, BMI, WHR, alcohol consumption, smoking status, 4 diet-related variables, and 8 health-related variables) and six in men (BMI, recent weight loss, smoking status, commuting by cycle, DASH score, and frequency of standing at work; Fig. 2a and Supplementary Figure S4). Among these, birth weight was considered a potential confounder in women, whereas the remaining variables were treated as potential mediators.

**Fig. 2:**
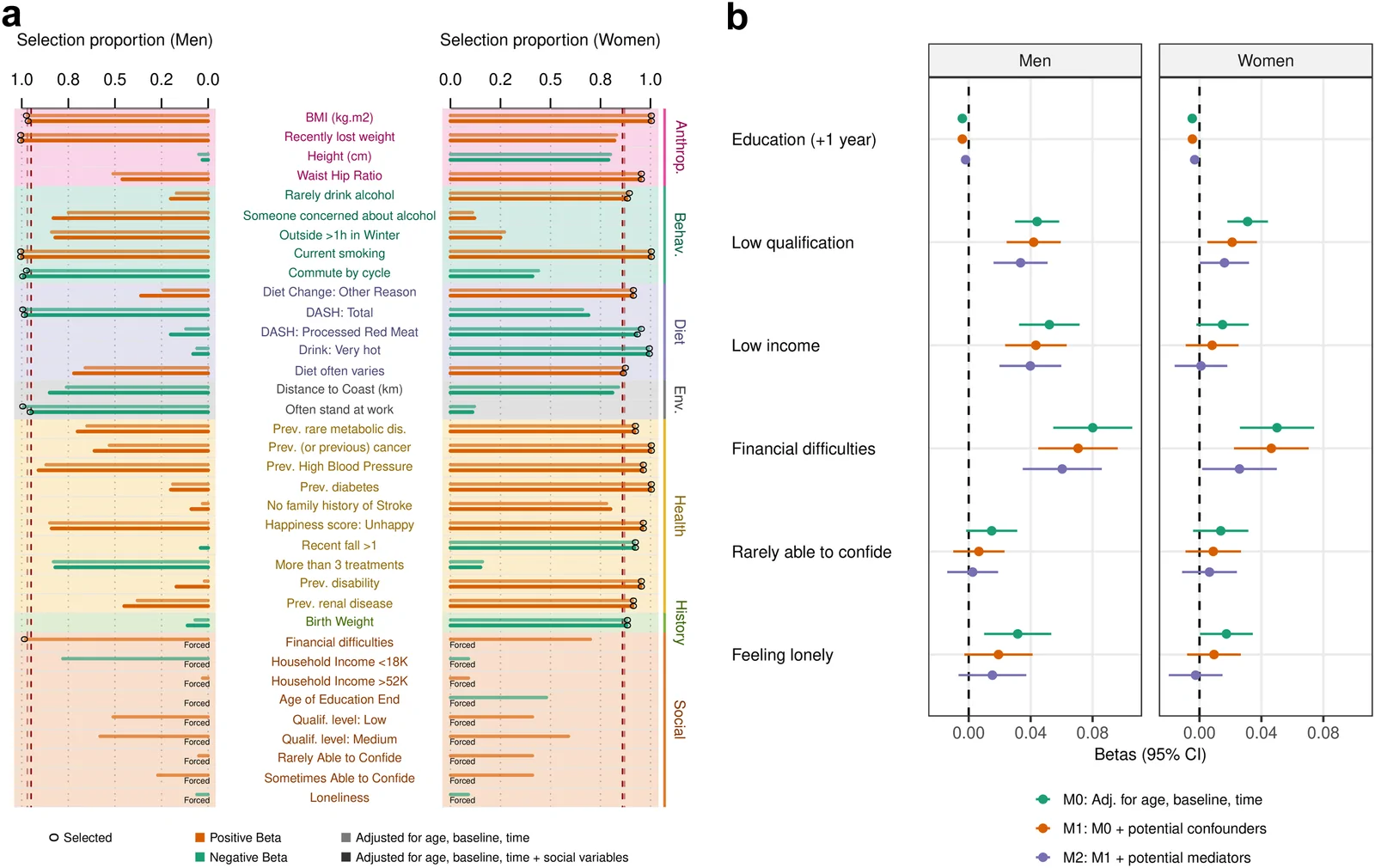
**Determinants of changes in Biological Health Score among UK Biobank participants with repeat assessments (United Kingdom, mean follow-up 4.3 years).** (a) Selection proportions of candidate predictors retained in at least 80% of subsamples in at least one sex stratum and of social factors of interest, based on a multivariate stability selection procedure using LASSO regression models stratified by sex. All models were adjusted for age, baseline BHS, and time between the two assessments, and were either additionally adjusted for the six social factors of interest (dark bars) or not (lighter bars). Bars are coloured red when the estimated coefficients were positive and green when negative. Predictors retained in the final model are indicated by a circle at the end of the corresponding bar. (b) Forest plot displaying regression coefficients (β) and 95% confidence intervals for the associations between each social factor and change in BHS, estimated using linear regression models stratified on sex. Model 0 was adjusted for age, baseline BHS, and time between measurements. Model 1 was additionally adjusted for previously selected confounders and social factors considered causally prior to the exposure of interest, providing an estimate of the exploratory total effect of each social factor on BHS change. Model 2 was further adjusted for previously selected mediators and social factors considered causally subsequent to the exposure of interest, providing an estimate of the exploratory direct effect. For example, in plot b, a positive coefficient indicates that the corresponding social factor is associated with a greater increase in BHS over the 4-year period, and attenuation between Model 1 and Model 2 suggests that the association may be partly explained by downstream behavioural, dietary, anthropometric, or health-related factors.

In models adjusted for age, baseline BHS, time between assessments, selected confounders and social factors considered causally prior to the exposure of interest, educational and economic disadvantages were associated with BHS change in both women and men (Fig. 2b). Specifically, each additional year of education was negatively associated with BHS change in women (−0.005, 95% Confidence Interval (CI) −0.007, −0.003) and men (−0.004, 95% CI −0.006, −0.002). Similarly, having a low qualification was associated with higher BHS change in women (0.021, 95% CI 0.005, 0.037) and men (0.042, 95% CI 0.025, 0.059). Financial difficulties were also associated with higher BHS change in both women (0.046, 95% CI 0.022, 0.071) and men (0.071, 95% CI 0.045, 0.096). Low income was associated with higher BHS change in men (0.043, 95% CI 0.024, 0.063) but not in women. Social engagement, including rarely being able to confide in someone and feeling lonely, were not associated with BHS change after adjustment for potential confounders. Further adjustment for the variables identified as potential mediators attenuated most associations. The association with education was markedly reduced in both sexes and was no longer observed in men, whereas associations with low qualification and financial difficulties remained significant.

Across sub-scores (Supplementary Figure S6), economic factors showed the most consistent exploratory total effect. Financial difficulties were associated with higher changes in the metabolic, inflammatory, and renal sub-scores in both sexes, and with changes in the cardiovascular sub-score in men. Socio-affective factors (loneliness and rarely being able to confide) showed little evidence of association (only loneliness with inflammatory sub-score in men). Education was associated with inflammatory and renal changes, while low qualification was mainly associated with the cardio sub-score. Analyses using the original multi-category coding of educational attainment, household income and ability to confide showed similar patterns of association with BHS change (Supplementary Figure S5). In particular, low and medium educational attainment showed similar associations relative to high educational attainment, as did the two highest household income categories relative to the lowest (<£18,000).

### Association between changes in the BHS and health outcomes

Age-scaled Cox models, adjusted for baseline BHS and time between the assessments (models M0) indicate an overall increased risk of all-cause mortality, AS-CVD (with stronger effects observed in men) and cancer incidence (Fig. 3, and Supplementary Table S5a, b and c). Schoenfeld residual plots were not indicative of a violation of the proportional hazards assumption for any of the outcome of interest both in men and women (results not shown). We found that an increase in BHS was significantly associated with higher all-cause mortality with an HR of 1.46 (95% CI 1.25, 1.71) in men and 1.26 (95% CI 1.02, 1.56) in women; with higher risk of AS-CVD incidence with an HR of 1.33 (95% CI 1.13, 1.57) in men and 1.24 (95% CI 1.03, 1.48) in women, and a higher risk of cancer incidence with an HR of 1.29 (95% CI 1.06, 1.58) in women and 1.16 (95% CI 1.01, 1.41) in men.

**Fig. 3:**
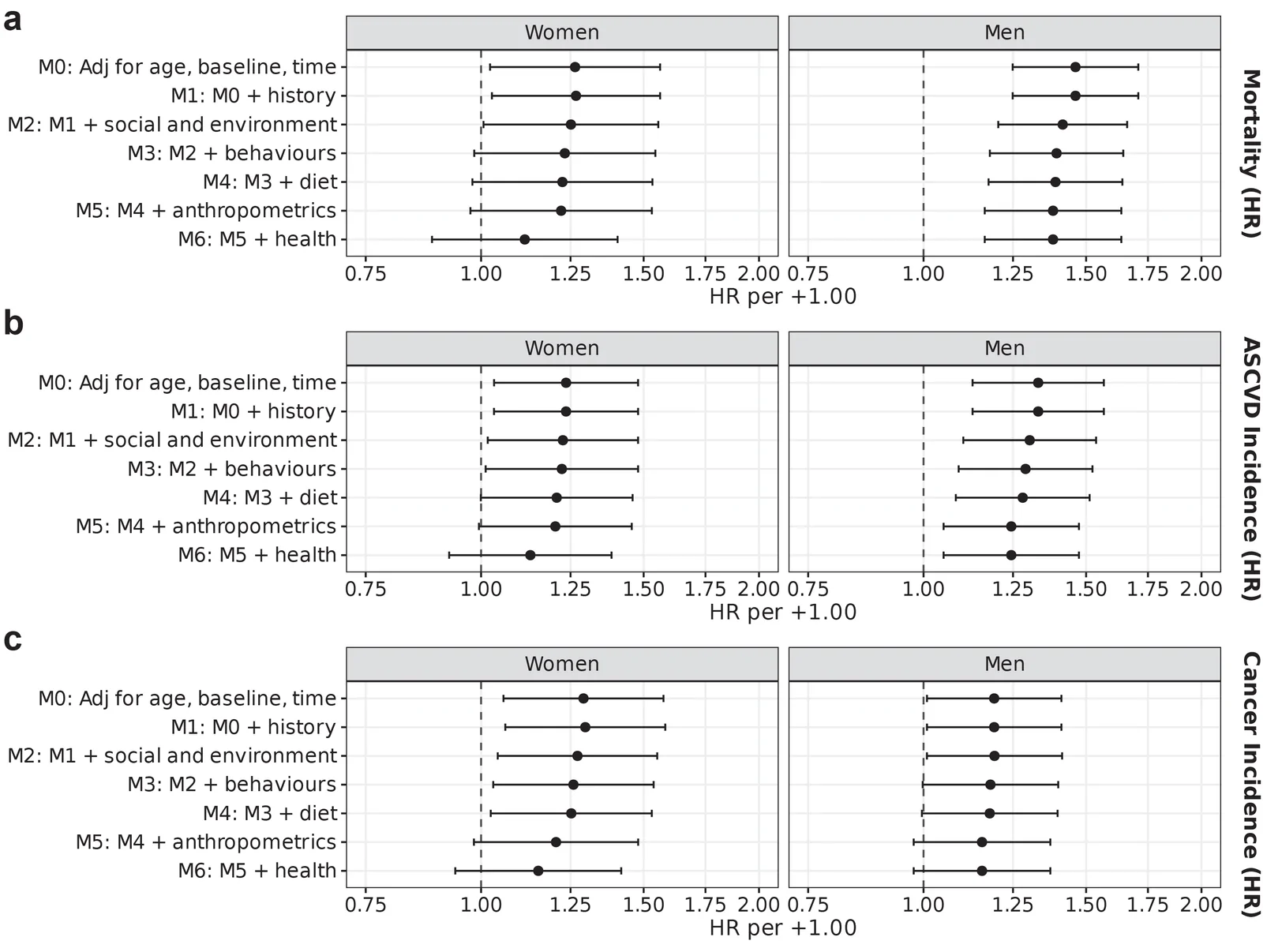
**Hazard ratios and 95% confidence intervals for a +1.0 increase in BHS change score.** Multivariate Cox proportional hazard regression models of (a) cancer incidence, (b) atherosclerotic cardiovascular disease (AS-CVD) incidence and (c) all-cause mortality among UK Biobank participants with repeat biomarker assessments conducted approximately four years apart in the United Kingdom using age as timescale, stratified on sex and adjusted for baseline BHS and time between 2 assessments (M0) and sequentially adjusted for history-related variables (M1), socioeconomic, work and environmental factors (M2), behaviours (M3), diet (M4), anthropometric (M5), and health variables (M6), as identified through the multivariate variable selection procedure performed for each sex. Follow-up for health outcomes started at the second assessment visit and ended in 2022, December 6th. For example, a hazard ratio above 1 indicates that a greater increase in BHS is associated with a higher subsequent risk of the outcome. Changes in hazard ratios across models M0 to M6 illustrate the influence of additional and sequential adjustment for social, behavioural, dietary, anthropometric and health-related variables.

Our sequential adjustment analyses showed a slight effect attenuation across all health outcomes and both sexes, with the strongest attenuation observed in women while adjusting for health variables. In women, the fully adjusted effects of the change in BHS on all-cause mortality, AS-CVD and incidence are no longer statistically significant (HR being 1.12, 95% CI 0.89, 1.41; 1.13, 95% CI 0.92, 1.39 and 1.16. 95% CI 0.94–1.42, respectively). In men, the fully adjusted effects of the change in BHS on all-cause mortality and AS-CVD incidence remains statistically significant (HR being 1.38, 95% CI 1.17, 1.64 and 1.25, 95% CI 1.05–1.47, repsectively) but not for cancer (1.16, 95% CI 0.98–1.37).

Analysis exploring each sub-score separately (Supplementary Figure S7) suggest that the association with mortality seems mainly driven by the cardiovascular and inflammatory systems in women and the inflammatory, liver and renal systems in men; the association with AS-CVD, by inflammatory, metabolic and renal system in men and inflammatory and liver system in women; and the association with cancer by the inflammatory and renal system in women and renal system in men.

### Mediated part by BHS changes of the effect of social determinant on health

Sex-specific counterfactual Total Effects (TE, Fig. 4 and Supplementary Tables S7), based on Cox regression models, indicated that one less year of education (TE = 1.04, 95% CI 1.02, 1.07), low income (1.51, 95% CI 1.27, 1.80]) and financial difficulties (1.45, 95% CI 1.09, 1.96]) were associated with higher all-cause mortality in men and one less year of education (TE = 1.05, 95% CI 1.00, 1.09) in women. One less year of education has also a significant total effect on AS-CVD incidence in both men and women (respectively TE = 1.05, 95% CI 1.04, 1.08] and 1.08 (95% CI 1.03, 1.10) and financial difficulties on cancer incidence in women (TE = 1.45, 95% CI 1.12, 1.87).

**Fig. 4:**
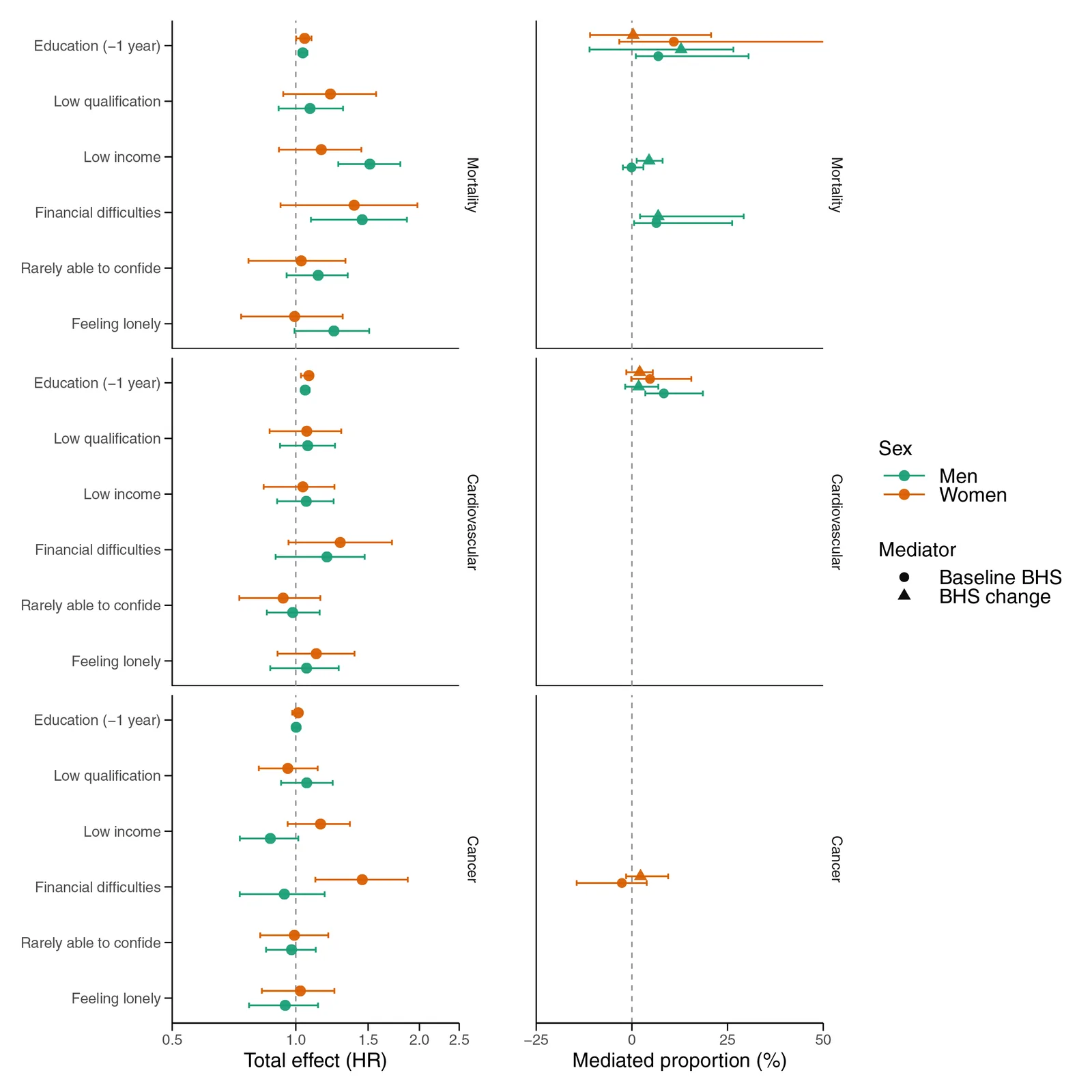
**Mediation analyses among UK Biobank participants with repeat biomarker assessments (United Kingdom, baseline assessment 2006–2010, repeat assessment approximately 4 years later).** Results of mediation analyses examining the role of baseline BHS and BHS change in the association between social disadvantage and subsequent cancer incidence, AS-CVD incidence, and all-cause mortality among UK Biobank participants aged 37–73. Total effects of age of education end (one less year), low/medium qualification (vs. high), low household income (<18 k vs. more), rarely able to confide in someone (vs. sometimes, always) and feeling lonely (vs. no) are given in the multiplicative scale in the first column. For each social exposure, if the total effect is significant, mediated proportions (PM) by baseline BHS and BHS change score are given (in percentage of total effect) in the second column. For example, the first column shows the total association between each social exposure and the outcome. When this total effect was statistically significant (i.e. TE of low income on mortality among men), the second column shows the proportion of this association mediated by baseline BHS or BHS change. A positive mediated proportion indicates that higher BHS or greater BHS increase accounts for part of the association between social disadvantage and the health outcome.

For each of these significant total effects, we estimated the proportion that was mediated through BHS at baseline and BHS changes. In men, baseline BHS explained 6.89% (95% CI 1.04, 30.48) of the effect of education, 6.38% (95% CI 0.56, 26.20) of the effect of financial difficulties on all-cause mortality; and 8.34% (95% CI 3.50, 18.55) of the effect of education on AS-CVD incidence. BHS change explain the effect of economic factors on mortality in men: 4.48% (95% CI 1.25, 8.02) of income total effect and 6.87% (95% CI 2.12, 29.23) of the total effect of financial difficulties. We did not observed any significant mediated proportion for women.

Sensitivity analyses using alternative definition of qualification, income and ability to confide (Supplementary Table S8) yielded similar results except for the proportion mediated of low income by BSH change on mortality, which was no longer significant and the Total Effect of low qualification on AS-CVD which became significant in women.

## Discussion

This study extends previous work on cross-sectional multisystem biological scores by investigating their short-term changes using repeated biomarker measurements and their contribution to the pathways linking social exposures to major health outcomes. The BHS and its 4-year change were derived from 15 biomarkers representing five physiological systems. The availability of repeated biomarker measurements allows the investigation their short-term trajectories: individuals with similar baseline BHS values may experience very different trajectories over time, and these may carry important prognostic information. The collection of outcomes via linkage continued through the end of 2022, allowing for a follow-up period of 9–10 years after the second measurement.

We found that BHS increased over time irrespective of sex, consistent with the cumulative nature of multisystem physiological dysregulation. All sub-scores increased between the two measurements except the cardiovascular sub-score. This finding raises questions about the relevance of cardiovascular markers for capturing physiological wear-and-tear. More than half of participants reported taking medication, and nearly one in five were receiving antihypertensive treatment.^30^ In terms of prospective events, pharmacological control of blood pressure may represent a form of physiological adaptation, meaning that controlled blood pressure under treatment may be functionally similar to untreated low blood pressure. However, when studying determinants of physiological dysregulation, such treatment effects may reduce the sensitivity of cardiovascular biomarkers to capture underlying deterioration.

The *cumulative* nature of the score suggests that its progression is not merely a consequence of chronological ageing but may be intensified by repeated or severe exposures, leading to accelerated physiological wear-and-tear. In our study, changes in the BHS were associated with socioeconomic conditions independently of age and baseline BHS level, suggesting that the score reflects part of the cumulative physiological consequences or traces of socially-patterned processes of exposure embodiment. The strong association between BHS progression and baseline health variables, especially in women, raises the question of whether BHS change reflects a causal mechanism in the embodiment process or primarily serves as a proxy for underlying health status.

Survival analyses revealed that a 4-year increase in BHS was associated with higher incidence of AS-CVD and, cancer, and higher all-cause mortality, independently of age and BHS value at baseline. Even after adjusting for social factors, diet, behaviours, anthropometric measures, and health at baseline, the association with AS-CVD and mortality remained statistically significant in men. This is consistent with other studies conducted in other populations and using other methods for measuring biological scores and/or changes in these scores.^10^^,^^31^^,^^32^ These findings suggest that BHS changes may capture risk-related information not fully accounted for by measured baseline social, behavioural, anthropometric, and health-related factors.

Associations with mortality and health outcomes were observed independently of baseline BHS, suggesting that trajectories of multisystem physiological dysregulation may provide prognostic information beyond static measures. This perspective is consistent with theoretical frameworks of embodiment, which conceptualise physiological dysregulation as a dynamic process accumulating over time rather than a static biological state.^16^^,^^17^ Our findings therefore suggest that the dynamics of multisystem physiological dysregulation may provide complementary information to single time-point measures, capturing evolving biological vulnerability that is socially-patterned and predictive of later health outcomes.

In mediation analyses, we found limited evidence that short-term changes in BHS may explain the association between social disadvantage and later health outcomes. Most mediated proportions through BHS change were not statistically significant. However, small but significant mediated proportions were observed for low income (around 4%) and financial difficulties (around 7%) in relation to all-cause mortality in men. Even when statistically significant, mediated proportions remained small, indicating that multiple other pathways are likely to contribute. This suggests that short-term changes in multisystem dysregulation capture only part of the longer-term biological embodiment of social experiences.

These results should also be interpreted in light of the modelling strategy. Although the mediation framework is robust and allows the consideration of intermediate confounding, such analyses require substantial statistical power. Despite the large sample size, the number of events for several outcomes remained limited, which may have reduced the ability to detect total effects of some social exposures. Furthermore, although a strength of our approach is that covariate selection was conducted across a broad set of candidate variables using a stability-selection framework—thereby reducing arbitrariness in model building and limiting overfitting—this procedure was designed primarily to improve adjustment strategies and reduce model specification bias rather than to identify a definitive set of causal determinants or explanatory variables. Residual confounding, but also measurement error, and imperfect classification of selected variables as confounders or mediators remain possible. Similarly, although medication information contributed to the definition of several prevalent health conditions, we cannot exclude treatment-related influences on biomarker trajectories independent of the underlying physiological processes of interest. Additional concerns, including reverse causality, lack of consistency across outcomes, and potential selection bias, further warrants a cautious interpretation of these findings as definitively causal. Moreover, short-term changes in multisystem physiological dysregulation may be insufficient to capture the full biological consequences of social disadvantage, particularly for diseases with long latency periods such as cancers.

Our study leveraged the large sample size and high-quality data of the UK Biobank. Even after restricting the analysis to participants with two biomarker measurements, the cohort included thousands of individuals with validated follow-up data from clinical registries, enabling robust investigation of mortality and disease incidence. Our analysis also benefited from an extensive set of candidate behavioural, environmental, and health-related variables, allowing a systematic data-driven identification of factors associated with BHS change. However, the UK Biobank is known to exhibit a healthy-volunteer selection bias,^33^ here amplified by the inclusion of participants with two measures, which affects the generalisability of our findings.

Our study has several limitations. We used a broad definition of cancer, encompassing multiple sites. The clinical heterogeneity of cancer types and the lower cancer incidence observed in the UK Biobank compared with the general population^33^ may explain why our findings for cancer were weaker. Specifically, combining cancers with potentially distinct latency periods and etiological pathways may have diluted associations that could be more apparent for specific cancer groups such as smoking-related or obesity-related cancers. Furthermore, the relatively short interval between biomarker measurements may be less suited to capturing biological processes involved in cancers with long latency periods.

In addition, the BHS was calculated using 15 biomarkers representing five physiological systems. While there is ongoing debate regarding the optimal scoring method and no consensus on how to measure these scores over time, sensitivity analyses using alternative scoring approaches (not presented here) yielded consistent conclusions. Although some biomarkers included in the BHS are correlated, particularly within physiological systems, they capture complementary aspects of systems included in the BHS. Alternative approaches based on latent variables or dimensionality reduction techniques may provide complementary information and would deserve further investigation.

Change score analysis has been challenged in the literature; however, as established by Tennant et al., analyses of change scores that adjust for the baseline value (when the baseline value is a mediator) can produce unbiased estimates of the direct effect of the exposure on the change, as this is mathematically equivalent to follow-up adjusted for baseline value.^34^ The use of a change score rather than the follow-up value is justified here because this change is considered, throughout the study, not only as an outcome but also as an exposure and a mediator.

Overall, we found that greater increases in BHS over four years were associated with higher risks of mortality, cancer and AS-CVD independently of baseline levels, which had previously been shown to predict these outcomes.^15^ Importantly, these associations persisted after adjustment for behavioural, dietary, anthropometric, and health factors. However, we found limited evidence that short-term changes in BHS substantially mediate the association between social disadvantage and health outcomes.

In conclusion, we showed that 4-year change in a previously validated composite biological score, independently of age and baseline level, is socially patterned and associated with subsequent all-cause mortality, cancer and AS-CVD incidence, with weaker evidence for cancer. Although BHS change provided only modest gains in prediction and explained only a small proportion of the association between social disadvantage and later mortality, it appears to capture an evolving multisystem physiological vulnerability relevant to later health. Together, these findings suggest that repeated biological measurements may provide a way into physiological signatures of embodiment processes over the life course. Extending these analyses using several (more than two) measurements throughout the lifecourse may represent an important follow-up study exploring the dynamics of the exposure-driven biological changes and how these may affect health and ageing.

## Contributors

HC: Conceptualisation, data curation, formal analysis, methodology, visualisation, writing (original draft), writing (review and editing). RW, RCZ: formal analysis, methodology, visualisation, writing (review and editing); DT: data curation, formal analysis, visualisation; LN, EHF, RC: results interpretation, writing (review and editing); SG, TW: Conceptualisation, writing (review and editing), funding acquisition; TS, MK-I, CD: Conceptualisation, writing (review and editing), funding acquisition, supervision, project administration; DV, MC-H: Conceptualisation, methodology, investigation, project administration, writing (original draft), writing (review and editing), supervision. HC, RW, RCZ, DV, and MC-H accessed and verified the underlying data all authors were responsible for the decision to submit the manuscript.

## Data sharing statement

This study was conducted using the UK Biobank resource under application number 69328 granting access to the corresponding UK Biobank biomarkers, and phenotype data. No additional data available. UK Biobank data is publicly avail-able upon application on the UK biobank website (https://www.ukbiobank.ac.uk/register-apply/).

## Declaration of interests

MC-H holds shares of the O-SMOSE company. Consulting activities of the company are independent of the present work. MKI has received funding from the European Research Council through the Gender and Health Inequalities (GENDHI) project (grant agreement 856478) for work that is unrelated to the present research. The other authors have no interest to declare.
