# Supplementary material for: Contribution of short-term changes in a composite health score on pathways linking social disadvantage and health: evidence from the UK biobank prospective cohort study
Source: eClinicalMedicine. 2026 Sep 29;100:104221. doi: 10.1016/j.eclinm.2026.104221 (PMC13654542; doi:10.1016/j.eclinm.2026.104221)
Supplement: Supplementary file 1 [file mmc1.pdf]

Supplementary - Tables

Table S1. Outcomes definitions

ICD-10, ICD-9, OPCS-4 describe the codes used to identify cases through consented linkage to the national death registers and NHS central registers (cancer and hospital registers). 6150, 20001, 20002, 20003, 20004 are the UKBB field used to identify prevalent cases at baseline through questionnaire.

Variable	Description	ICD-10	ICD-9	OPCS-4	6150	20001	20002	20003 (ATC)	20004
**Outcomes**									
**CVD: Atherosclerotic**	**Ischaemic heart diseases (angor, myocardial infarction...), diseases of arteries, arterioles and capillaries (including peripheral artery disease) and ischaemic stroke**	I20–I25, I63, I65–I66, I70–I73	410–414, 433–437, 440–443	K40–K46, K49–K50, K75, L14–L16	2		1074–1075, 1081–1082, 1087–1088, 1583	–	1070, 1071, 1095, 1102–1105, 1107–1110, 1555
**CANCER: All**	**Malignant neoplasms and neoplasms of uncertain or unknown behaviour**	C00–C43, C45–C97, D37–D48	140–172, 174–209, 235–239	X70–X73, X65	–	1001–1088	–	L01–L02	1595, 1625

Table S2. Exposures definitions

Variable	UKBB Field	Comments	% NA
Age at the end of education	845	If missing, age was computed using Field 22501 (year of education end) and 34 (year of birth)	15.8
Qualification level	6138	High (College or University degree), Medium (A levels/AS levels or equivalent, NVQ or HND or HNC or equivalent, Other professional qualifications), Low (O levels/GCSEs or equivalent, CSEs or equivalent)	10.5
Household income	738	Regrouped in 3 categories: "Less than 18K" (0:1), "18K to 52K" (2:3) and "More than 52K" (4:5)	10.9
Financial difficulties	6145	Yes if "Financial difficulties" (code 6) experienced in the last 2 years	1.8
Being able to confide	2110	Regrouped in 3 categories: "Yes" (4:5), "Sometimes" (2:3) and "Rarely" (0:1)	2.6
Feeling lonely	2020	“Yes”, “No”	1.3

Table S3a. Type, group and number of covariates

	Group		Variable	Type	Categories
	.Base		Demo_Sex	binary	Men; Women
	.Base		Demo_Age	quantitative	
1	Anthrop	1	Anthrop_Waist_cm	quantitative	
2	Anthrop	2	Anthrop_Hip_cm	quantitative	
3	Anthrop	3	Anthrop_Height_cm	quantitative	
4	Anthrop	4	Anthrop_Weight_kg	quantitative	
5	Anthrop	5	Anthrop_FacialAge	categorical	About your age; Younger than you are; Older than you are
6	Anthrop	6	Anthrop_ComparedWeight	categorical	Gained; Same; Lost
7	Anthrop	7	Anthrop_BMI_kgm2	quantitative	
8	Anthrop	8	Anthrop_WHR	quantitative	
9	Behav	1	Behav_ActPhy_FreqVig	categorical	2.Moderate; 3.Regurarly; 1.None
10	Behav	2	Behav_ActPhy_FreqMod	categorical	1.Some; 2.Daily; 0.Never
11	Behav	3	Behav_ActPhy_FreqWalk	categorical	2.Moderate; 3.Daily; 1.Rarely
12	Behav	4	Behav_ActPhy_Tot	categorical	2.Intermediate; 3.Active; 0.Sedentary; 4.Sportperson
13	Behav	5	Behav_ActPhy_METscore	quantitative	NA
14	Behav	6	Behav_Smok_Status	categorical	Current; Never; Previous
15	Behav	7	Behav_Smok_AgeStart	categorical	2.Before 17; 1.Never started; 3.After 18
16	Behav	8	Behav_Smok_Passive	binary	Yes; No
17	Behav	9	Behav_Alc_Frequency	categorical	2.Rarely; 3.1_or2aWeek; 4.3_or4aWeek; 5.Daily_orAlmost; 0.AlwaysNever; 1.CurrentlyNever
18	Behav	10	Behav_Alc_AmountADay	categorical	4.MoreThan7; 1.1or2; 2.3or4; 1.None; 3.5or6
19	Behav	11	Behav_Alc_Change10Y	categorical	1.Less; 3.More; 2.Same; 0.Never
20	Behav	12	Behav_Alc_DrinkBeer	categorical	2.MoreThanOnceaWeek; 0.No; 1.OnceaWeek
21	Behav	13	Behav_Alc_DrinkFortWine	categorical	0.No; 2.MoreThanOnceaWeek; 1.OnceaWeek
22	Behav	14	Behav_Alc_DrinkRedWine	categorical	2.MoreThanOnceaWeek; 0.No; 1.OnceaWeek
23	Behav	15	Behav_Alc_DrinkSpirit	categorical	0.No; 2.MoreThanOnceaWeek; 1.OnceaWeek
24	Behav	16	Behav_Alc_DrinkWhiteWine	categorical	0.No; 1.OnceaWeek; 2.MoreThanOnceaWeek
25	Behav	17	Behav_Alc_EverInjured	binary	No; Yes
26	Behav	18	Behav_Alc_SbConcern	binary	No; Yes
27	Behav	19	Behav_Alc_More6aDay	categorical	2.Regularly; 0.No; 1.Rarely
28	Behav	20	Behav_Alc_TotWeek_Cat	categorical	3.13+_aWeek; 1.1to5_aWeek; 2.6to12_aWeek; 0.0_aWeek
29	Behav	21	Behav_Alc_Addict	categorical	No; Former; Current
30	Behav	22	Behav_Addic_Drugs	categorical	No; Former; Current
31	Behav	23	Behav_Addic_CannabisEver	categorical	2.Yes; 0.Never; 1.Tested
32	Behav	24	Behav_Addic_Behav	categorical	No; Former; Current
33	Behav	25	Behav_Transp_DriveFast	categorical	2.Often; 0.No; 1.Sometimes
34	Behav	26	Behav_Transp_VehicNb	categorical	2.2ormore; 1.One; 0.None
35	Behav	27	Behav_Transp_DriveTime	categorical	2.2ormore; 0.None; 1.1hour
36	Behav	28	Behav_Transp_TypeMotor	binary	Yes; No
37	Behav	29	Behav_Transp_TypeWalk	binary	No; Yes
38	Behav	30	Behav_Transp_TypePublic	binary	No; Yes
39	Behav	31	Behav_Transp_TypeCycle	binary	No; Yes
40	Behav	32	Behav_Other_UseSunProtect	categorical	1.Often; 0.Rarely; 2.Always
41	Behav	33	Behav_Other_MobilUse	categorical	2.MoreThan5Y; 0.Never; 1.Recently
42	Behav	34	Behav_Other_MobilFreqWeek	categorical	1.MoreThan30min; 0.Rarely; 1.LessThan30min
43	Behav	35	Behav_Other_ComputerFreqDay	categorical	1.1hour; 2.MoreThan2H; 0.Rarely
44	Behav	36	Behav_Other_TVFreqDay	categorical	1.2hours; 2.MoreThan3H; 0.Rarely
45	Behav	37	Behav_Other_OutsideWin	categorical	2.MoreThan1H; 1.1hour; 0.Rarely
46	Behav	38	Behav_Other_OutsideSum	categorical	1.3to4hour; 2.MoreThan5H; 0.LessThan3H
47	Behav	39	Behav_Other_RiskTaking	binary	Yes; No
48	Behav	40	Behav_LeisureAct_Gym	binary	No; Yes
49	Behav	41	Behav_LeisureAct_Pub	binary	No; Yes
50	Behav	42	Behav_LeisureAct_Religious	binary	No; Yes
51	Behav	43	Behav_LeisureAct_Adulteduc	binary	No; Yes
52	Behav	44	Behav_LeisureAct_Other	binary	No; Yes
53	Diet	1	Diet_Change5Y	categorical	0.Nochange; 2.ChangeOtherReason; 1.ChangeForIllness
54	Diet	2	Diet_Variation	categorical	1.Sometimes; 0.Rarely; 2.Often
55	Diet	3	Diet_IntakeBeef	categorical	1.OnceaWeek; 0.Never,rarely; 2.SeveralTimesaWeek
56	Diet	4	Diet_IntakePoultry	categorical	2.SeveralTimesaWeek; 1.OnceaWeek; 0.Never,rarely
57	Diet	5	Diet_IntakeLamb	categorical	0.Never,rarely; 1.OnceaWeek; 2.SeveralTimesaWeek
58	Diet	6	Diet_IntakePork	categorical	0.Never,rarely; 1.OnceaWeek; 2.SeveralTimesaWeek
59	Diet	7	Diet_IntakeProcessedMeat	categorical	2.SeveralTimesaWeek; 0.Never,rarely; 1.OnceaWeek

60	Diet	8	Diet_IntakeBreadW	categorical	1.LessThan10; 2.MoreThan10; 0.Rarely
61	Diet	9	Diet_IntakeCerealW	categorical	1.LessThan6; 2.MoreThan6; 0.Rarely
62	Diet	10	Diet_IntakeCheese	categorical	0.Never,rarely; 2.SeveralTimesaWeek; 1.OnceaWeek
63	Diet	11	Diet_IntakeSpreadType	categorical	1.Butter; 2.Other; 0.NoSpread
64	Diet	12	Diet_IntakeMilkType	categorical	3.SemiSkimmedMilk; 2.SkimmedMilk; 4.FullcreamMilk; 0.NoMilk; 1.NoCowMilk
65	Diet	13	Diet_IntakeFreshFruitD	categorical	1.LessThan2; 0.Rarely; 2.MoreThan2
66	Diet	14	Diet_IntakeNonOilyFish	categorical	0.Never,rarely; 2.SeveralTimesaWeek; 1.OnceaWeek
67	Diet	15	Diet_IntakeOilyFish	categorical	0.Never,rarely; 1.OnceaWeek; 2.SeveralTimesaWeek
68	Diet	16	Diet_IntakeCookedVegetD	categorical	1.LessThan3; 2.MoreThan3; 0.Rarely
69	Diet	17	Diet_IntakeRawVegetD	categorical	0.Rarely; 1.LessThan2; 2.MoreThan2
70	Diet	18	Diet_IntakeSalt	categorical	1.Sometimes; 0.Rarely; 2.Often
71	Diet	19	Diet_IntakeTeaD	categorical	1.LessThan4; 2.MoreThan4; 0.Rarely
72	Diet	20	Diet_IntakeCoffeeType	categorical	0.Other; 2.Ground; 0.NoCoffee; 1.Instant
73	Diet	21	Diet_IntakeWaterD	categorical	1.LessThan3; 0.Rarely; 2.MoreThan3
74	Diet	22	Diet_TemperatureDrink	categorical	1.Hot; 0.NotorWarm; 2.VeryHot
75	Diet	23	Diet_DASH_Fruit	quantitative	1; 2; 3; 4; 5
76	Diet	24	Diet_DASH_Lowfat.dairy	quantitative	1; 2; 3; 4
77	Diet	25	Diet_DASH_Processed.red.meat	quantitative	1; 2; 3; 4
78	Diet	26	Diet_DASH_Vegetables	quantitative	1; 2; 3; 4; 5
79	Diet	27	Diet_DASH_Whole.grain	quantitative	1; 2; 3; 4; 5
80	Diet	28	Diet_DASH_Dash	quantitative	1; 2; 3; 4; 5
81	Diet	29	Diet_NeverEatEgg	binary	No; Yes
82	Diet	30	Diet_NeverEatSugar	binary	No; Yes
83	Env	1	Work_Exp_Abestos	categorical	0.No; 1.Sometimes; 2.Often
84	Env	2	Work_Exp_Chemical	categorical	1.Sometimes; 0.No; 2.Often
85	Env	3	Work_Exp_Cold	categorical	0.No; 1.Sometimes; 2.Often
86	Env	4	Work_Exp_Diesel	categorical	0.No; 1.Sometimes; 2.Often
87	Env	5	Work_Exp_Dust	categorical	0.No; 1.Sometimes; 2.Often
88	Env	6	Work_Exp_Hot	categorical	0.No; 1.Sometimes; 2.Often
89	Env	7	Work_Exp_Noise	categorical	0.No; 1.Sometimes; 2.Often
90	Env	8	Work_Exp_Paints	categorical	1.Sometimes; 0.No; 2.Often
91	Env	9	Work_Exp_Pesticide	categorical	0.No; 1.Sometimes; 2.Often
92	Env	10	Work_Exp_Physical	categorical	0.No; 2.Often; 1.Sometimes
93	Env	11	Work_Exp_Shift	binary	No; Yes
94	Env	12	Work_Exp_Smoke	categorical	2.Often; 0.No; 1.Sometimes
95	Env	13	Work_Exp_Stand	categorical	1.Sometimes; 0.No; 2.Often
96	Env	14	Work_TimeTravelWeek	categorical	2.MoreThanOnceaDay; 1.OnceaDay; 0.Never
97	Env	15	Work_TimeTravelDist	categorical	2.MoreThan10km; 1.2to10km; 0.LessThan1Miles
98	Env	16	Work_TimeWork	categorical	2.MoreThan40hours; 0.LessThan30hours; 1.30to40hours
99	Env	17	Env_Scotland	binary	England_Wales; Scotland
100	Env	18	Env_DistToCoast_Km	quantitative	
101	Env	19	Env_Traffic_CloseMajorRoad	binary	No; Yes
102	Env	20	Env_Traffic_IntensMajorRoad_VhDay	quantitative	
103	Env	21	Env_Traffic_IntensNearRoad_VhDay	quantitative	
104	Env	22	Env_Traffic_TotIntensRoads_VhDay	quantitative	
105	Env	23	Env_Traffic_DistNearRoad_invM	quantitative	
106	Env	24	Env_Traffic_LenghtMajorRoad_Mtr	quantitative	
107	Env	25	Env_DomesticGarden300_Perc	quantitative	
108	Env	26	Env_GreenSpace300_Perc	quantitative	
109	Env	27	Env_NaturEnv300_Perc	quantitative	
110	Env	28	Env_WaterSpace300_Perc	quantitative	
111	Env	29	Env_NitroDiox_2010_ugm3	quantitative	
112	Env	30	Env_NitroOx_2010_ugm3	quantitative	
113	Env	31	Env_NoisePoll_DayTime_Db	quantitative	
114	Env	32	Env_AirPoll_PM10_ugm3	quantitative	
115	Env	33	Env_AirPoll_PM07_ugm3	quantitative	
116	Env	34	Env_AirPoll_PM25_ugm3	quantitative	
117	Env	35	Env_RuralOrUrban	binary	Urban; Rural
118	Env	36	Home_Exp_CookWithGas	binary	Yes; No
119	Env	37	Home_Exp_HeatGasFire	binary	No; Yes
120	Env	38	Home_Exp_HeatSolidFuelFire	binary	No; Yes
121	Health	1	Health_General_LongStandingIllness	binary	No; Yes
122	Health	2	Health_General_OverallScore	categorical	1.Good; 0.Excellent; 2.FairPoor
123	Health	3	Health_General_HealthHappiness	categorical	0.Happy; 1.Moderate; 2.Unhappy
124	Health	4	Health_General_TreatmentNB	categorical	0.None; 2.TwoorThree; 1.One; 3.MoreThan3

125	Health	5	Health_General_RecentFall	categorical	0.None; 1.One; 2.MoreThan1
126	Health	6	Health_General_AttendanceAllowance	binary	No; Yes
127	Health	7	Health_General_DisabilityAllowance	binary	No; Yes
128	Health	8	Health_General_BlueBadge	binary	No; Yes
129	Health	9	Health_General_HandGripMain	quantitative	NA
130	Health	10	Health_General_HandGripOther	quantitative	NA
131	Health	11	Health_Sympt_Fainting	binary	No; Yes
132	Health	12	Health_Sympt_ChestDiscomfort	binary	No; Yes
133	Health	13	Health_Sympt_ChestWhistling	binary	Yes; No
134	Health	14	Health_Pain_HeadFace	binary	No; Yes
135	Health	15	Health_Pain_BackNeck	binary	Yes; No
136	Health	16	Health_Pain_Abdo	binary	No; Yes
137	Health	17	Health_Pain_HipKnee	binary	No; Yes
138	Health	18	Health_Other_BowelKScreen	categorical	0.Never; 2.Recently; 1.NotRecently
139	Health	19	Health_AUTRE_ALLERGIE_Prev	binary	No; Yes
140	Health	20	Health_AUTRE_AUTOIMM_Prev	binary	No; Yes
141	Health	21	Health_AUTRE_DERMATO_Prev	binary	No; Yes
142	Health	22	Health_AUTRE_HEMACHRON_Prev	binary	No; Yes
143	Health	23	Health_AUTRE_INFLAMCHRON_Prev	binary	No; Yes
144	Health	24	Health_AUTRE_METABRARE_Prev	binary	No; Yes
145	Health	25	Health_CVD_AF_Prev	binary	No; Yes
146	Health	26	Health_CVD_ASCVD_Prev	binary	No; Yes
147	Health	27	Health_CVD_CHD_Prev	binary	No; Yes
148	Health	28	Health_CVD_HBP_Prev	binary	No; Yes
149	Health	29	Health_CVD_IS_Prev	binary	No; Yes
150	Health	30	Health_CVD_MI_Prev	binary	No; Yes
151	Health	31	Health_CVD_PAD_Prev	binary	No; Yes
152	Health	32	Health_CVD_Valv_Prev	binary	No; Yes
153	Health	33	Health_CVD_VTE_Prev	binary	No; Yes
154	Health	34	Health_ENDO_DT_Prev	binary	No; Yes
155	Health	35	Health_ENDO_THYR_Prev	binary	No; Yes
156	Health	36	Health_INJURY_OTHER_Prev	binary	No; Yes
157	Health	37	Health_INJURY_SEVERE_Prev	binary	No; Yes
158	Health	38	Health_PELV_GYANDRO_Prev	binary	No; Yes
159	Health	39	Health_PELV_LITH_Prev	binary	No; Yes
160	Health	40	Health_PELV_RENAL_Prev	binary	No; Yes
161	Health	41	Health_PELV_URO_Prev	binary	No; Yes
162	Health	42	Health_PSY_ANX_Prev	binary	No; Yes
163	Health	43	Health_PSY_MOOD_Prev	binary	No; Yes
164	Health	44	Health_PSY_TOT_Prev	binary	No; Yes
165	Health	45	Health_NEURO_TOT_Prev	binary	No; Yes
166	Health	46	Health_INFECT_TOT_Prev	binary	No; Yes
167	Health	47	Health_HANDICAP_TOT_Prev	binary	No; Yes
168	Health	48	Health_CANCER_Prev	binary	No; Yes
169	Health	49	Health_DIGEST_TOT_Prev	binary	No; Yes
170	Health	1	History_Birth_Weight	quantitative	NA
171	Health	2	History_Birth_Caesarian	binary	No; Yes
172	Health	3	History_Birth_MultBirth	binary	No; Yes
173	Health	4	History_Birth_MatSmok	binary	No; Yes
174	Health	5	History_Birth_BrestFed	binary	No; Yes
175	Health	6	History_Child_RecurrATB	binary	No; Yes
176	Health	7	History_Child_BodySize10	categorical	Thinner; About_average; Plumper
177	Health	8	History_Child_HeigthSize10	categorical	Taller; About_average; Shorter
178	Health	9	History_Child_ACEFeltHated	categorical	No; Sometimes; Often
179	Health	10	History_Child_ACEFeltLoved	categorical	Sometimes; Yes; No
180	Health	11	History_Child_ACEPhysAbuse	categorical	No; Sometimes; Often
181	Health	12	History_Child_ACESexMolest	categorical	No; Sometimes; Often
182	History	13	History_Child_ACESbCare	categorical	Yes; No; Sometimes
183	History	14	History_Fam_Adopted	binary	No; Yes
184	History	15	History_Fam_Brother	binary	Yes; No
185	History	16	History_Fam_Sister	binary	Yes; No
186	History	17	History_Fam_BioFatherDeathYoung	binary	Yes; No
187	History	18	History_Fam_BioMotherDeathYoung	binary	No; Yes
188	History	19	Stress_Events_BellitByPartner	binary	Yes; No
189	History	20	Stress_Events_PhyViolentPartner	binary	No; Yes
190	History	21	Stress_Events_SexWoConsentPartner	binary	No; Yes

191	History	22	Stress_Events_LifeThreatAccident	binary	No; Yes
192	History	23	Stress_Events_ExposedToWar	binary	No; Yes
193	History	24	Stress_Events_LifeThreatIllness	binary	Yes; No
194	History	25	Stress_Events_VictimViolence	binary	No; Yes
195	History	26	Stress_Events_WitnSuddenDeath	binary	Yes; No
196	History	27	Stress_Events_VictimSexAssault	binary	No; Yes
197	History	28	Stress_Events_PersonnalIllness	binary	No; Yes
198	History	29	Stress_Events_RelativeIllness	binary	No; Yes
199	History	30	Stress_Events_RelativeDeath	binary	No; Yes
200	History	31	Stress_Events_Separation	binary	No; Yes
201	History	32	Stress_PTSD_Tot	binary	Yes; No
202	History	33	Health_Family_HeartDis	binary	No; Yes
203	History	34	Health_Family_Stroke	binary	No; Yes
204	History	35	Health_Family_HighBP	binary	Yes; No
205	History	36	Health_Family_ChronicBronch	binary	No; Yes
206	History	37	Health_Family_Alzheimer	binary	No; Yes
207	History	38	Health_Family_Diabetes	binary	No; Yes
208	History	39	Health_Family_Parkinson	binary	No; Yes
209	History	40	Health_Family_SevereDepression	binary	No; Yes
210	History	41	Health_Family_LungCancer	binary	No; Yes
211	History	42	Health_Family_BowelCancer	binary	Yes; No
212	History	43	Health_Family_BreastCancer	binary	No; Yes
213	History	44	Health_Family_ProstateCancer	binary	No; Yes
214	Mental	1	Mental_General_Happiness	categorical	1.Moderate; 0.VeryHappy; 2.Unhappy
215	Mental	2	Mental_General_MeaningfulLife	categorical	1.Moderate; 0.VeryMuch; 2.NotReally
216	Mental	3	Mental_General_ProfHelp	binary	Yes; No
217	Mental	4	Mental_General_GPSeen	binary	Yes; No
218	Mental	5	Mental_General_PsySeen	binary	Yes; No
219	Mental	6	Mental_General_ImpactfulDistress	binary	No; Yes
220	Mental	7	Mental_Personality_Neuroticism	quantitative	
221	Mental	8	Mental_Personality_MoodswingsN12	binary	Yes; No
222	Mental	9	Mental_Personality_MiserablenessN12	binary	Yes; No
223	Mental	10	Mental_Personality_IrritavilityN12	binary	No; Yes
224	Mental	11	Mental_Personality_SensitivityN12	binary	Yes; No
225	Mental	12	Mental_Personality_FedUpN12	binary	Yes; No
226	Mental	13	Mental_Personality_NervousN12	binary	No; Yes
227	Mental	14	Mental_Personality_WorryN12	binary	Yes; No
228	Mental	15	Mental_Personality_TenseN12	binary	No; Yes
229	Mental	16	Mental_Personality_EmbarassmentN12	binary	Yes; No
230	Mental	17	Mental_Personality_SufferNerveN12	binary	No; Yes
231	Mental	18	Mental_Personality_GuiltyN12	binary	Yes; No
232	Mental	19	Mental_Depression_SelfHarm	categorical	1.DeathToughts; 0.NeverDeathToughts; 3.SuicideAttempt; 2.SelfHarmed
233	Mental	20	Mental_Depression_RDS4Score	quantitative	
234	Mental	21	Mental_Depression_PQH9Score	quantitative	
235	Mental	22	Mental_Bipolar_ManiacoDep	binary	Yes; No
236	Mental	23	Mental_Anxiety_GAD7Score	quantitative	
237	Mental	24	Mental_Sleep_Duration	categorical	0.Between7to9H; 2.LessThan7hours; 1.MoreThan9H
238	Mental	25	Mental_Sleep_GettingUp	binary	Easy; NotEasy
239	Mental	26	Mental_Sleep_Chronotype	binary	Morning; Evening
240	Mental	27	Mental_Sleep_HavingNap	categorical	0.Never; 1.Sometimes; 2.Usually
241	Mental	28	Mental_Sleep_Insomnia	categorical	1.Sometimes; 0.Never; 2.Usually
242	Mental	29	Mental_Sleep_Snoring	binary	No; Yes
243	Mental	30	Mental_Sleep_Narcolepsy	categorical	0.Never; 2.Often; 1.Sometimes
244	Mental	31	Mental_Sleep_Tiredness	categorical	1.ALittle; 0.NotAtAll; 2.ALot
245	Mental	32	Mental_Sleep_Troubles	categorical	1.ALittle; 0.NotAtAll; 2.ALot
246	Mental	33	Mental_Cognitive_FluidIntell	quantitative	
247	Mental	34	Mental_Cognitive_MemoryMax	quantitative	
248	Mental	35	Mental_Depression_Ever	binary	Yes; No
249	Mental	36	Mental_Bipolar_Ever	binary	No; Yes
250	Mental	37	Mental_Anxiety_Ever	binary	Yes; No
251	Mental	38	Mental_Psychose_Ever	binary	No; Yes
252	SocioEco	1	Demo_Ethnicity_AreaBirth	categorical	Scotland; England; Wales; Asian country; African country; Northern Ireland; European country; North American country; South American country; Republic of Ireland

253	SocioEco	2	Demo_Ethnicity_Background	categorical	White, Bristish; Asian; White, Other; White, Irish; Mixed, Black; Black; Other; Mixed, Other
254	SocioEco	3	Demo_Ethnicity_SkinColor	categorical	VeryFair_Fair; LightOlive_DarkOlive; Brown_Black
255	SocioEco	4	SocioEco_Eco_EmployStatus	categorical	Paid; Retired; StayAtHome; Unemployed; Disability; Other
256	SocioEco	5	SocioEco_Eco_EmployGaps_Nb	categorical	0.None; 1.One; 2.Two_ormore
257	SocioEco	6	SocioEco_Eco_AblePayRent	categorical	Yes; No; Often
258	SocioEco	7	SocioEco_Eco_AccomOwner	categorical	Own with a mortgage; Own outright; No
259	SocioEco	8	SocioEco_Eco_AccomSameAdress	categorical	2to5y; More10y; Less2y; 6to10y
260	SocioEco	9	SocioEco_Eco_AccomType	categorical	House; Flat; Other
261	SocioEco	10	SocioEco_Eco_GapsSickness	binary	No; Yes
262	SocioEco	11	SocioEco_Eco_GapsUnemploy	binary	No; Yes
263	SocioEco	12	SocioEco_Eco_GapsStayAtHome	binary	No; Yes
264	SocioEco	13	SocioEco_Eco_GapsUnpaidWork	binary	No; Yes
265	SocioEco	14	SocioEco_Eco_GapsSmallJobs	binary	No; Yes
266	SocioEco	15	SocioEco_Social_FriendVisFreq	categorical	1.Onceaweek; 0.SeveralTimesaWeek; 2.LessThanOnceaWeek
267	SocioEco	16	SocioEco_Social_ConfindingRelation	categorical	Sometimes; Yes; Rarely
268	SocioEco	17	SocioEco_Social_ChildrenNb_class	categorical	3.Three_ormore; 2.Two; 1.One; 0.No
269	SocioEco	18	SocioEco_Social_LiveWith_Class	categorical	1.OnlyPartner; 2.PartnerAndChildren; 0.Alone; 3.OnlyChildren; 4.Other
270	SocioEco	19	SocioEco_ScoreEnv_IMD	quantitative	
	Exposure		SocioEco_Educ_AgeEnd	quantitative	
	Exposure		SocioEco_Educ_QualifLev_Cat	categorical	Medium; High; Low
	Exposure		SocioEco_Eco_HouseIncome	categorical	18kto52k; More52k; Less18k
	Exposure		SocioEco_Eco_FinancialDiff	binary	No; Yes
	Exposure		SocioEco_Social_AbleConfide	categorical	Rarely; Always; Sometimes
	Exposure		SocioEco_Social_Loneliness	binary	No; Yes

Tables S3b. Biomarkers, missing data

	T1		T2	
	**N**	**(%)**	**N**	**(%)**
**Cholesterol LDL (mmol/L)**	1369	6.83	2469	12.31
**Cholesterol HDL (mmol/L)**	2798	13.95	4590	22.89
**Triglycerides (mmol/L)**	1322	6.59	2463	12.28
**HBA1c (mmol/mol)**	1646	8.21	6025	30.05
**Systolic Blood pressure (mmHg)**	587	2.93	58	0.29
**Diastolic Blood pressure (mmHg)**	587	2.93	57	0.28
**Pulse Rate (bpm)**	587	2.93	57	0.28
**CRP (mg/L)**	1349	6.73	2471	12.32
**Glycoprotein Acetyls (mmol/L)**	2211	11.03	3466	17.29
**IGF-1 (nmol/L)**	1424	7.1	2607	13
**AST (U/L)**	1380	6.88	2503	12.48
**ALT (U/L)**	1316	6.56	2444	12.19
**GGT (U/L)**	1317	6.57	2445	12.19
**Creatinine (µmol/L)**	1323	6.6	2465	12.29
**Cystatin (mg/L)**	1316	6.56	2444	12.19

Tables S4. Changes in biomarkers, overall and by sex

For each biomarker, the table reports the standard deviation (SD) of the baseline measurement (T1), as well as the mean change between the two assessments (T2–T1), calculated separately for women, men, and the total sample. The last column expresses the mean change as a percentage of the baseline SD, providing a standardized indication of the magnitude of change relative to the variability of the biomarker at baseline.

	Women			Men			Total		
	SD (T1)	Mean (T2-T1)	%SD	SD (T1)	Mean (T2-T1)	%SD	SD (T1)	Mean (T2-T1)	%SD
**Cholesterol LDL (mmol/L)**	0.86	0.07	**8.51**	0.85	-0.06	**-7.64**	0.86	0.01	**0.66**
**Cholesterol HDL (mmol/L)**	0.37	-0.09	**-24.73**	0.31	-0.08	**-25.64**	0.38	-0.09	**-22.73**
**Triglycerides (mmol/L)**	0.88	0.1	**11.92**	1.11	-0.05	**-4.68**	1.02	0.03	**2.77**
**HBA1c (mmol/mol)**	5.29	0.83	**15.75**	6.00	0.77	**12.77**	5.65	0.8	**14.17**
**Systolic Blood pressure (mmHg)**	18.69	2.67	**14.27**	16.91	-0.1	**-0.62**	18.09	1.32	**7.27**
**Diastolic Blood pressure (mmHg)**	9.78	-0.81	**-8.33**	9.60	-2.02	**-21.04**	9.88	-1.4	**-14.2**
**Pulse Rate (bpm)**	10.11	-1.29	**-12.75**	11.36	-1.7	**-15**	10.78	-1.49	**-13.83**
**CRP (mg/L)**	4.34	-0.09	**-2.10**	4.00	0.18	**4.39**	4.18	0.04	**0.94**
**Glycoprotein Acetyls (mmol/L)**	5.67	0.72	**12.66**	5.28	0.46	**8.7**	5.5	0.59	**10.75**
**IGF-1 (nmol/L)**	0.12	0.02	**13.88**	0.12	0.01	**9.65**	0.12	0.01	**11.81**
**AST (U/L)**	8.33	0.86	**10.37**	8.96	0.05	**0.5**	8.8	0.46	**5.28**
**ALT (U/L)**	12.13	0.43	**3.53**	13.47	-1.08	**-8.00**	13.18	-0.31	**-2.32**
**GGT (U/L)**	29.18	0.72	**2.47**	36.74	-0.92	**-2.51**	33.67	-0.08	**-0.24**
**Creatinine (µmol/L)**	11.03	2.08	**18.85**	13.95	2.9	**20.78**	15.2	2.48	**16.31**
**Cystatin (mg/L)**	0.14	0.03	**19.81**	0.14	0.03	**21.01**	0.14	0.03	**19.86**

Tables S5. Survival analysis

Table S5a. Results of the survival and attenuation analysis with **mortality** as outcome. “Variable” is the exposure of interest (change score or sub-scores), and for each model we describe the hazard ratios (HR), 95% confidence intervals (CI), p-value (p) and C-index for an increase of +1.0.

	Women			Men		
**Model**	**HR [95% CI]**	**p**	**C-index**	**HR [95% CI]**	**p**	**C-index**
M0. Baseline + TBV	1.264 [1.023; 1.563]	0.0302	0.582	1.461 [1.249; 1.708]	2.11E-06	0.572
M1. + History	1.267 [1.027; 1.563]	0.0269	0.585	1.461 [1.249; 1.708]	2.11E-06	0.572
M2. + Socio.	1.251 [1.006; 1.556]	0.0439	0.598	1.415 [1.205; 1.662]	2.31E-05	0.600
M3. + Behaviours	1.232 [0.983; 1.544]	0.0699	0.605	1.393 [1.180; 1.646]	9.29E-05	0.616
M4. + Diet	1.225 [0.979; 1.533]	0.0765	0.628	1.390 [1.176; 1.642]	0.000111	0.616
M5. + Anthropo.	1.221 [0.974; 1.531]	0.0836	0.630	1.381 [1.165; 1.638]	0.000202	0.621
M6. + Health	1.115 [0.885; 1.406]	0.3560	0.668	1.381 [1.165; 1.638]	0.000202	0.621

Table S5b. Results of the survival and attenuation analysis with **cancer** incidence as outcome. “Variable” is the exposure of interest (change score or sub-scores), and for each model we describe the hazard ratios (HR), 95% confidence intervals (CI), p-value (p) and C-index for an increase of +1.0.

	Women			Men		
**Model**	**HR [95% CI]**	**p**	**C-index**	**HR [95% CI]**	**p**	**C-index**
M0. Baseline + TBV	1.291 [1.058; 1.576]	0.0121	0.536	1.193 [1.009; 1.410]	0.0394	0.517
M1. + History	1.297 [1.062; 1.583]	0.0107	0.536	1.193 [1.009; 1.410]	0.0394	0.517
M2. + Socio.	1.272 [1.042; 1.552]	0.0178	0.553	1.194 [1.008; 1.413]	0.0396	0.533
M3. + Behaviours	1.259 [1.031; 1.538]	0.024	0.563	1.182 [0.998; 1.399]	0.0528	0.549
M4. + Diet	1.252 [1.024; 1.531]	0.0281	0.57	1.180 [0.996; 1.397]	0.0558	0.549
M5. + Anthropo.	1.206 [0.982; 1.480]	0.0736	0.577	1.157 [0.976; 1.372]	0.0933	0.551
M6. + Health	1.153 [0.938; 1.418]	0.177	0.61	1.157 [0.976; 1.372]	0.0933	0.551

Table S5c. Results of the survival and attenuation analysis with **atherosclerotic cardiovascular** disease incidence as outcome. “Variable” is the exposure of interest (change score or sub-scores), and for each model we describe the hazard ratios (HR), 95% confidence intervals (CI), p-value (p) and C-index for an increase of +1.0.

	Women			Men		
**Model**	**HR [95% CI]**	**p**	**C-index**	**HR [95% CI]**	**p**	**C-index**
M0. Baseline + TBV	1.236 [1.033; 1.479]	0.0207	0.576	1.331 [1.130; 1.568]	0.000616	0.572
M1. + History	1.236 [1.033; 1.479]	0.0208	0.576	1.331 [1.130; 1.568]	0.000616	0.572
M2. + Socio.	1.226 [1.017; 1.479]	0.0328	0.599	1.303 [1.104; 1.538]	0.00173	0.592
M3. + Behaviours	1.223 [1.011; 1.479]	0.0377	0.605	1.290 [1.092; 1.524]	0.0028	0.598
M4. + Diet	1.208 [0.999; 1.459]	0.0507	0.614	1.281 [1.084; 1.513]	0.00366	0.599
M5. + Anthropo.	1.204 [0.995; 1.456]	0.0567	0.619	1.245 [1.051; 1.474]	0.0111	0.604
M6. + Health	1.131 [0.924; 1.385]	0.234	0.664	1.245 [1.051; 1.474]	0.0111	0.604

Table S6. C-index according to BHS measures

C-index and confidence intervals (bootstrap, 200 replications) for age-scale cox models modelling mortality, atherosclerotic cardiovascular diseases (AS-CVD) and cancer by baseline BHS (V1 only), second measure of BHS (V2 only), baseline BHS and change score, adjusted on time between 2 assessment (V1 + change) as computed on the main analysis, baseline BHS and second measurement (V1+ V2), for men and women.

	Men			
	**V1 only**	**V2 only**	**V1 + change**	**V1 + V2**
**Mortality**	0.551 [0.527; 0.573]	0.560 [0.539; 0.584]	0.562 [0.540; 0.588]	0.557 [0.537; 0.584]
**AS-CVD**	0.559 [0.540; 0.578]	0.566 [0.546; 0.582]	0.572 [0.551; 0.590]	0.570 [0.550; 0.588]
**Cancer**	0.508 [0.496; 0.525]	0.505 [0.493; 0.522]	0.508 [0.498; 0.530]	0.508 [0.498; 0.529]
	**Women**			
	**V1 only**	**V2 only**	**V1 + change**	**V1 + V2**
**Mortality**	0.565 [0.536; 0.595]	0.565 [0.536; 0.598]	0.565 [0.537; 0.603]	0.568 [0.543; 0.605]
**AS-CVD**	0.569 [0.544; 0.596]	0.552 [0.528; 0.576]	0.569 [0.545; 0.597]	0.567 [0.542; 0.596]
**Cancer**	0.516 [0.497; 0.538]	0.528 [0.507; 0.548]	0.529 [0.511; 0.552]	0.528 [0.508; 0.552]

Tables S7. Mediation analysis

Results of the mediation analysis with **(a) mortality, (b) atherosclerotic cardiovascular disease** incidence and **(c) cancer** incidence as outcome. Total effects of age of education end (one less year), low/medium qualification (versus high), low income (<18k versus more), financial difficulties (yes versus no), ability to confide (rarely versus sometimes/always) and feeling lonely (yes versus no) are given in the multiplicative scale. For each social exposure, mediated proportions (PM) by baseline BHS and BHS change score are given (in percentage of total effect). Significant TE or PM are marked with a *.

	Women		Men	
	**Est.**	**95% IC**	**Est.**	**95% IC**
Deaths incidence at 5 years (%)	1.41		2.79	
**TE of 1 less year of education (multiplicative)**	**1.05**	**[1.00;1.09]***	**1.04**	**[1.02;1.07]***
PM of 1 less year of education via baseline BHS (%)	10.94	[-3.31;55.7]	6.89	[1.04;30.48]*
PM of 1 less year of education via BHS change (%)	0.32	[-10.94;20.69]	12.84	[-11.07;26.53]
**TE of a low qualification (multiplicative)**	**1.21**	**[0.93;1.57]**	**1.08**	**[0.91;1.30]**
PM of a low qualification via baseline BHS (%)	3.94	[-47.02;70.32]	25.59	[-235.66;214.18]
PM of a low qualification via BHS change (%)	1.47	[-11.18;18.98]	31.20	[-196.96;235.54]
**TE of a low income (multiplicative)**	**1.15**	**[0.91;1.44]**	**1.51**	**[1.27;1.80]***
PM of a low income via baseline BHS (%)	8.38	[-38.17;55.15]	-0.1	[-2.35;3.00]
PM of a low income via BHS change (%)	0.94	[-11.51;11.06]	4.48	[1.25;8.02]*
**TE of financial difficulties (multiplicative)**	**1.39**	**[0.92;1.98]**	**1.45**	**[1.09;1.86]***
PM of financial difficulties via baseline BHS (%)	14.38	[-76.87;84.31]	6.38	[0.56;26.20]*
PM of financial difficulties via BHS change (%)	2.02	[-14.60;19.71]	6.87	[2.12;29.23]*
**TE of unable to confide (multiplicative)**	**1.03**	**[0.77;1.32]**	**1.13**	**[0.95;1.34]**
PM of unable to confide via baseline BHS (%)	19.9	[-46.52;54.95]	0.56	[-19.81;16.12]
PM of unable to confide via BHS change (%)	36.22	[-13.7;014.01]	4.14	[-17.67;58.18]
**TE of feeling lonely (multiplicative)**	**0.99**	**[0.74;1.30]**	**1.24**	**[0.99;1.51]**
PM of feeling lonely via baseline BHS (%)	-28.91	[-62.38;49.30]	6.11	[-12.35;40.26]
PM of feeling lonely via BHS change (%)	-3.73	[-14.92;13.63]	2.58	[-6.42;21.21]

	Women		Men	
	**Est.**	**95% IC**	**Est.**	**95% IC**
AS-CVD incidence at 5 years (%)	2.86		5.40	
**TE of 1 less year of education (multiplicative)**	**1.08**	**[1.03;1.10]***	**1.05**	**[1.04;1.08]***
PM of 1 less year of education via baseline BHS (%)	4.73	[-0.18;15.52]	8.34	[3.50;18.55]*
PM of 1 less year of education via BHS change (%)	2.04	[-1.46;5.47]	1.77	[-1.76;6.90]
**TE of a low qualification (multiplicative)**	**1.06**	**[0.86;1.29]**	**1.07**	**[0.92;1.25]**
PM of a low qualification via baseline BHS (%)	9	[-64.18;86.97]	40.77	[-346.53;697.19]
PM of a low qualification via BHS change (%)	6.01	[-57.00;26.94]	27.48	[-199.78;155.42]
**TE of a low income (multiplicative)**	**1.04**	**[0.84;1.24]**	**1.06**	**[0.90;1.24]**
PM of a low income via baseline BHS (%)	10.44	[-42.38;62.63]	-0.52	[-62.38;62.56]
PM of a low income via BHS change (%)	-2.48	[-15.01;21.32]	13.2	[-109.67;135.94]
**TE of financial difficulties (multiplicative)**	**1.28**	**[0.96;1.71]**	**1.19**	**[0.89;1.47]**
PM of financial difficulties via baseline BHS (%)	13.12	[-57.24;57.79]	18.1	[-126.03;170.59]
PM of financial difficulties via BHS change (%)	2.31	[-28.91;21.73]	9.03	[-58.94;77.710]
**TE of unable to confide (multiplicative)**	**0.93**	**[0.73;1.15]**	**0.98**	**[0.85;1.14]**
PM of unable to confide via baseline BHS (%)	-1.81	[-34.30;33.12]	20.86	[-82.48;60.71]
PM of unable to confide via BHS change (%)	-1.53	[-15.14;14.60]	-4.73	[-38.87;38.49]
**TE of feeling lonely (multiplicative)**	**1.12**	**[0.90;1.39]**	**1.06**	**[0.87;1.27]**
PM of feeling lonely via baseline BHS (%)	-0.39	[-16.92;19.76]	24.25	[-137.35;159.93]
PM of feeling lonely via BHS change (%)	-0.25	[-9.44;7.62]	9.95	[-50.83;49.30]

	Women		Men	
	**Est.**	**95% IC**	**Est.**	**95% IC**
Cancer incidence at 5 years (%)	4.43		5.95	
**TE of 1 less year of education (multiplicative)**	**1.01**	**[0.98;1.04]**	**1.00**	**[0.98;1.02]**
PM of 1 less year of education via baseline BHS (%)	-10.52	[-204.72;145.09]	18.24	[-157.02;183.98]
PM of 1 less year of education via BHS change (%)	48.59	[-78.96;81.17]	188.64	[-95.13;75.24]
**TE of a low qualification (multiplicative)**	**0.96**	**[0.81;1.13]**	**1.06**	**[0.92;1.23]**
PM of a low qualification via baseline BHS (%)	5.78	[-61.77;68.63]	3.07	[-129.77;85.74]
PM of a low qualification via BHS change (%)	-5.73	[-33.14;40.82]	13.98	[-93.91;87.12]
**TE of a low income (multiplicative)**	**1.15**	**[0.96;1.35]**	**0.87**	**[0.73;1.01]**
PM of a low income via baseline BHS (%)	-1.45	[-16.37;7.50]	-0.01	[-3.13;2.8]
PM of a low income via BHS change (%)	2.04	[-12.03;19.93]	-2.87	[-21.01;5.98]
**TE of financial difficulties (multiplicative)**	**1.45**	**[1.12;1.87]***	**0.94**	**[0.73;1.18]**
PM of financial difficulties via baseline BHS (%)	-2.63	[-14.43;3.87]	-2.54	[-74.98;53.64]
PM of financial difficulties via BHS change (%)	2.26	[-1.52;9.47]	-11.3	[-89.84;116]
**TE of unable to confide (multiplicative)**	**0.99**	**[0.82;1.20]**	**0.98**	**[0.85;1.12]**
PM of unable to confide via baseline BHS (%)	17.35	[-34.54;24.94]	-0.07	[-12;15.72]
PM of unable to confide via BHS change (%)	-17.92	[-21.68;17.68]	-8.49	[-36.56;35.87]
**TE of feeling lonely (multiplicative)**	**1.03**	**[0.83;1.24]**	**0.94**	**[0.77;1.13]**
PM of feeling lonely via baseline BHS (%)	-6.86	[-22.43;32.72]	-1.77	[-45.34;45.58]
PM of feeling lonely via BHS change (%)	-2.31	[-18.22;18.63]	-2.87	[-19;18.32]

Tables S8. Mediation analysis with alternative definitions

Results of the mediation analysis with **(a) mortality, (b) atherosclerotic cardiovascular disease** incidence and **(c) cancer** incidence as outcome. Total effects of low qualification (versus medium/ high), low/medium income (<52k versus more) and ability to confide (rarely/sometimes versus always) are given in the multiplicative scale. For each social exposure, mediated proportions (PM) by baseline BHS and BHS change score are given (in percentage of total effect). Significant TE or PM are marked with a *.

	Women		Men	
	**Est.**	**95% IC**	**Est.**	**95% IC**
Deaths incidence at 5 years (%)	1.41		2.79	
**TE of a low qualification (multiplicative)**	**1.12**	**[0.87;1.44]**	**1.02**	**[0.85;1.21]**
PM of a low qualification via baseline BHS (%)	-2.93	[-33.31;38.42]	52.20	[-165.14;168.28]
PM of a low qualification via BHS change (%)	1.09	[-20.27;14.19]	-69.95	[-132.10;171.33]
**TE of a low income (multiplicative)**	**1.23**	**[0.90;1.71]**	**1.50**	**[1.21;1.88]***
PM of a low income via baseline BHS (%)	10.01	[-76.96;64.65]	0.34	[-1.46;3.06]
PM of a low income via BHS change (%)	0.36	[-6.07;6.73]	0.76	[-2.26;2.50]
**TE of unable to confide (multiplicative)**	**1.04**	**[0.78;1.33]**	**1.14**	**[0.95;1.35]**
PM of unable to confide via baseline BHS (%)	4.33	[-48.08;31.58]	-0.35	[-20.92;13.96]
PM of unable to confide via BHS change (%)	-2.49	[-20.09;14.17]	4.20	[-13.20;39.35]

	Women		Men	
	**Est.**	**95% IC**	**Est.**	**95% IC**
AS-CVD incidence at 5 years (%)	2.86		5.40	
**TE of a low qualification (multiplicative)**	**1.24**	**[1.02;1.48]***	**1.06**	**[0.92;1.23]**
PM of a low qualification via baseline BHS (%)	-0.21	[-6.11;5.53]	27.64	[-293.56;263.28]
PM of a low qualification via BHS change (%)	0.67	[-1.19;5.43]	10.08	[-73.45;86.51]
**TE of a low income (multiplicative)**	**1.2**	**[0.95;1.54]**	**1.15**	**[0.98;1.34]**
PM of a low income via baseline BHS (%)	6.01	[-32.49;49.64]	1.35	[-17.99;19.86]
PM of a low income via BHS change (%)	-0.16	[-6.77;7.53]	-0.71	[-8.31;8.75]
**TE of unable to confide (multiplicative)**	**0.96**	**[0.75;1.19]**	**0.98**	**[0.84;1.13]**
PM of unable to confide via baseline BHS (%)	3.06	[-35.4;34.16]	14.88	[-64.79;47.61]
PM of unable to confide via BHS change (%)	-3.76	[-10.87;15.14]	-8.95	[-47.1;51.32]

	Women		Men	
	**Est.**	**95% IC**	**Est.**	**95% IC**
Cancer incidence at 5 years (%)	4.43		5.95	
**TE of a low qualification (multiplicative)**	**0.92**	**[0.78;1.08]**	**1.03**	**[0.88;1.18]**
PM of a low qualification via baseline BHS (%)	0.13	[-15.95;17.03]	3.42	[-48.3;69.38]
PM of a low qualification via BHS change (%)	-2.83	[-27.34;24.73]	18.11	[-76.55;49.7]
**TE of a low income (multiplicative)**	**1.02**	**[0.85;1.26]**	**0.98**	**[0.84;1.14]**
PM of a low income via baseline BHS (%)	-27.3	[-101.61;89.03]	0.01	[-10.72;13.62]
PM of a low income via BHS change (%)	6.33	[-14.67;18.37]	1.21	[-16.5;22.55]
**TE of unable to confide (multiplicative)**	**0.98**	**[0.81;1.19]**	**0.96**	**[0.83;1.10]**
PM of unable to confide via baseline BHS (%)	0.56	[-21.98;21.41]	0.09	[-12.27;12.65]
PM of unable to confide via BHS change (%)	-18.37	[-15.33;19.68]	-5.39	[-33.4;31.01]

Supplementary - Figures

Figure S1. Data Analysis Flow chart

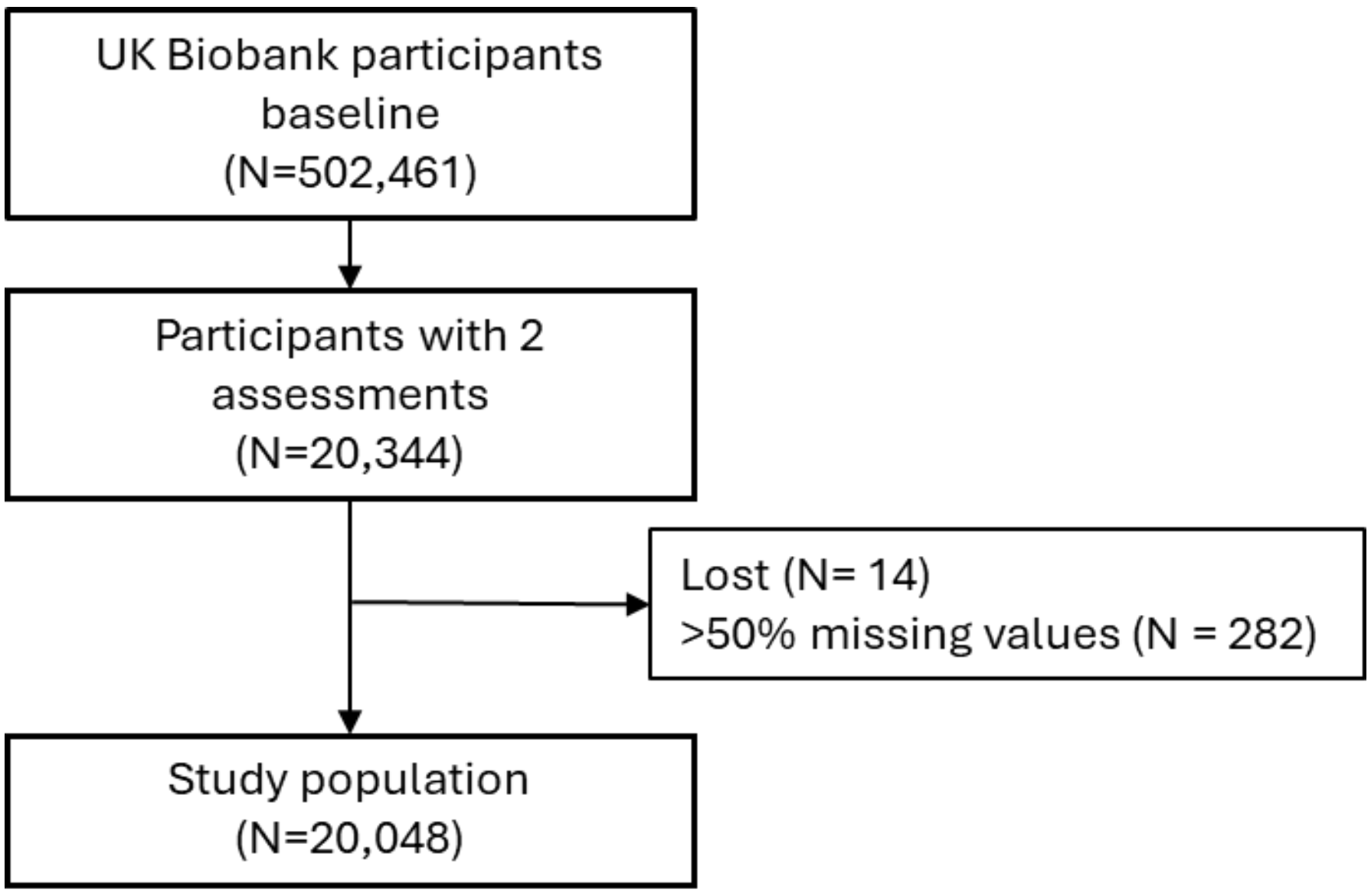

Figures S2. BHS changes over time

Figure S2a. Biological health score at baseline by age and sex (Local Polynomial Regression Fitting)

Figure S2b. Marginal means and confidence intervals by sex and BHS at baseline, from a linear regression modelling BHS change score by BHS at baseline, adjusted for time between 2 assessments and ethnicity and with an interaction term with age and sex.

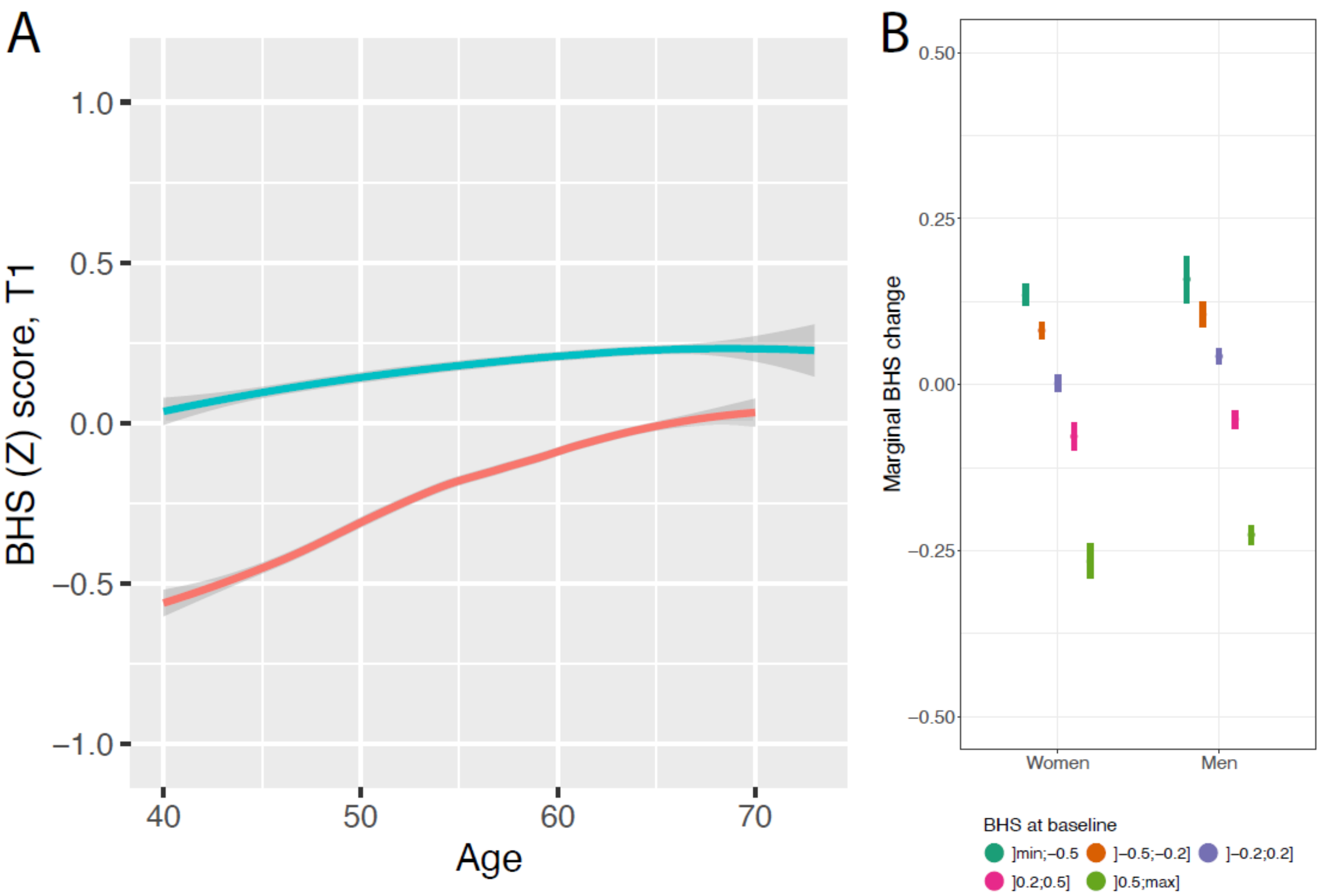

Figures S3. Distribution of changes in biomarkers between the two assessments (T2–T1) by sex

The distributions of changes in each biomarker, i.e., differences between the two measurements (T2–T1), are shown separately for women and men using kernel density curves. Curves are truncated to the range corresponding to ±1 standard deviations around the mean. Solid vertical lines indicate the mean change and dashed vertical lines indicate the median change for each sex. Colours correspond to sex.

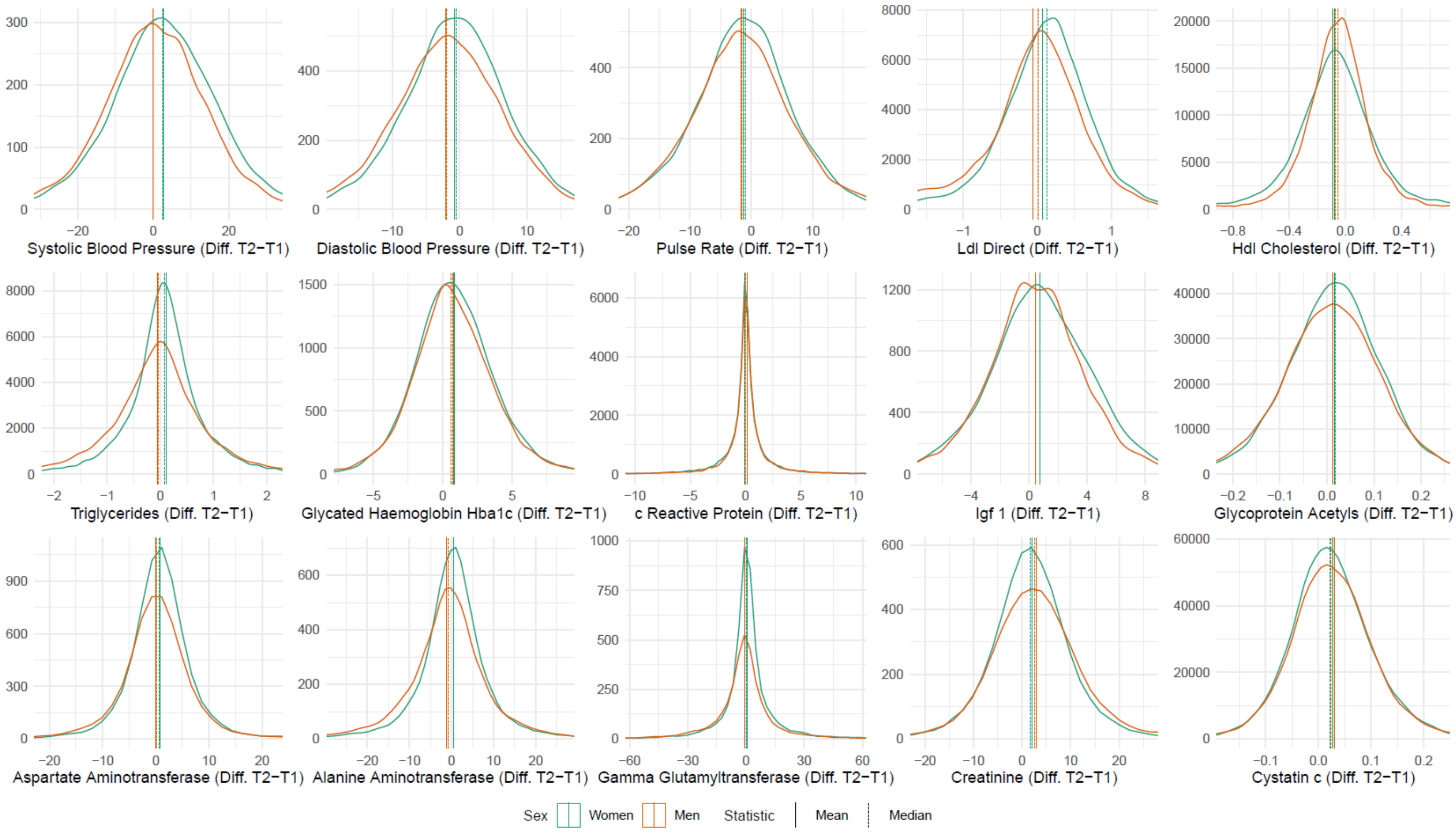

Figures S4. Determinants of BHS change (multivariate)

For each candidate determinants, selection proportions for men (y axis) and women (x axis) are describe in the first plot and the mean coefficient β (if the variable is selected at least once) for men (y axis) and women (x axis) in the second plot, from a multivariate stability selection approach using a series of LASSO regression models adjusted for age, BHS at baseline, time between 2 assessments and 6 social exposures, for each sex category. Predictors which are selected in the final model are represented by with larger circle and their labels are given. Colours represent the category of the determinants and numbers of variables selected in the final models in at least one sex category are given in the legend.

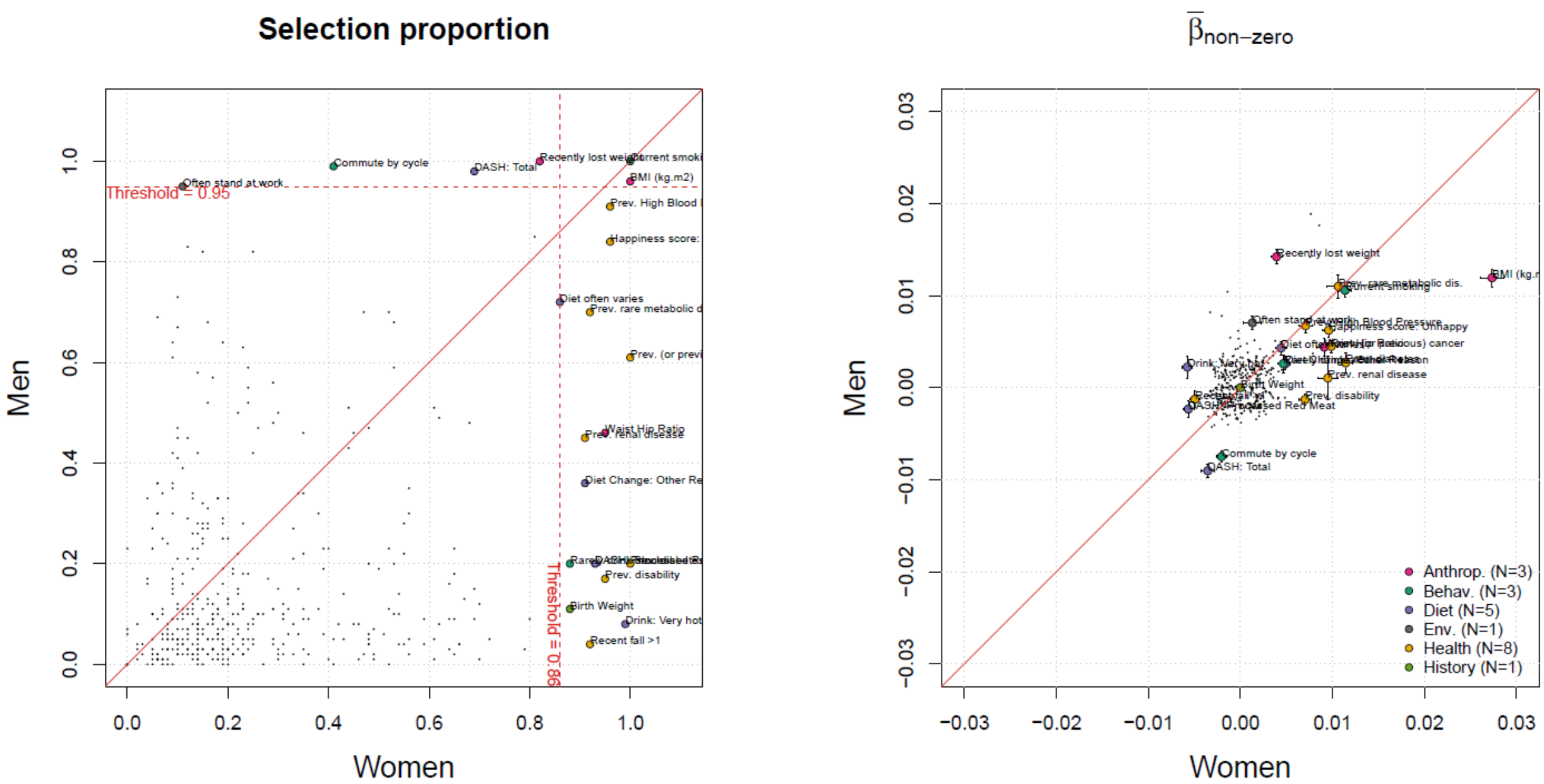

Figure S5. Forest plot for the associations between social factors (alternative definitions) and BHS change,

Forest plot displayng regression coefficients (β) and 95% confidence intervals for the associations between each social factor and change in BHS, estimated using linear regression models stratified on sex. Model 0 was adjusted for age, baseline BHS, and time between measurements. Model 1 was additionally adjusted for previously selected confounders and social factors considered causally prior to the exposure of interest, providing an estimate of the exploratory total effect of each social factor on BHS change. Model 2 was further adjusted for previously selected mediators and social factors considered causally subsequent to the exposure of interest, providing an estimate of the exploratory direct effect.

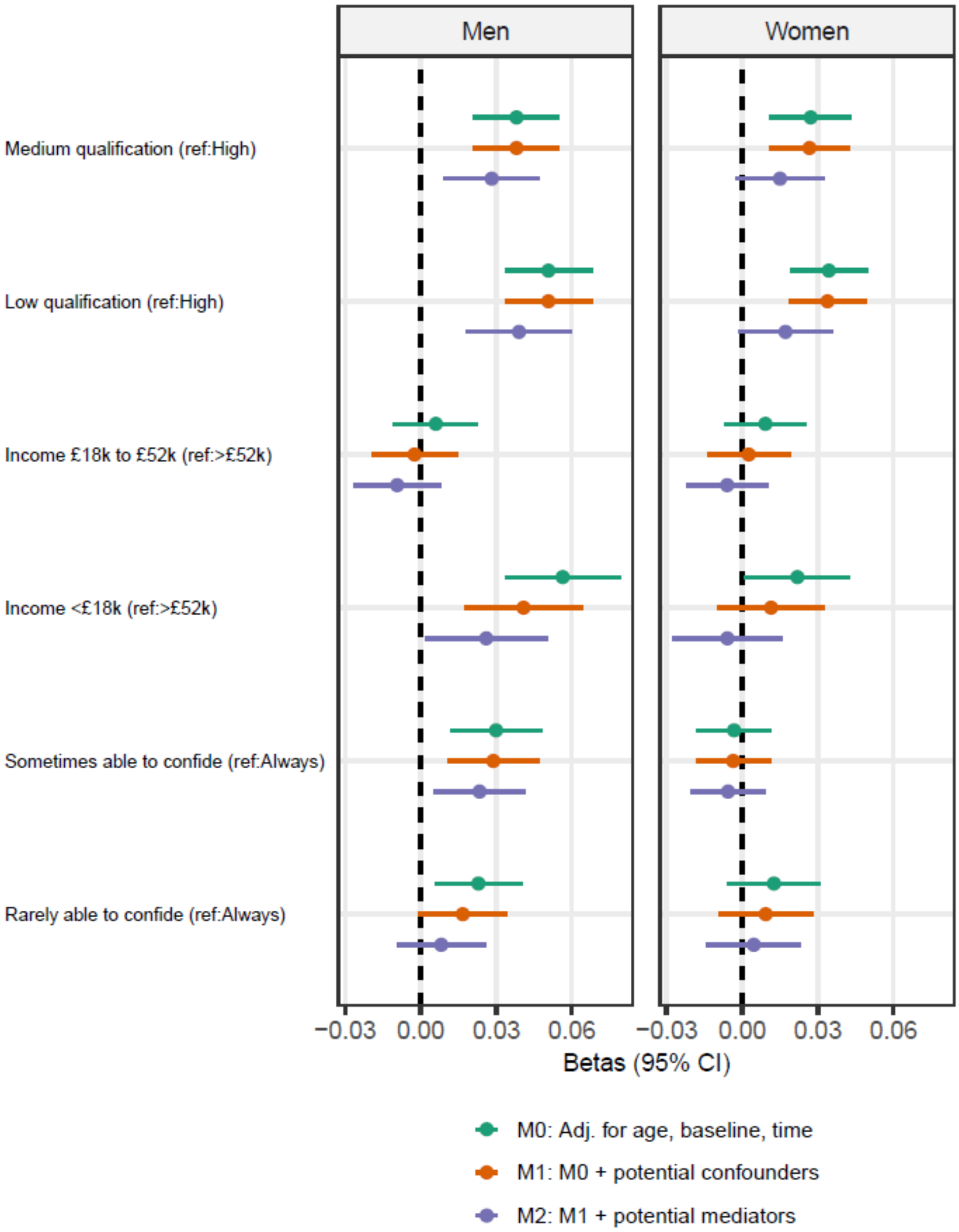

Figure S6. Forest plot for the associations between social factors and BHS change sub-scores,

Forest plot displaying regression coefficients (β) and 95% confidence intervals for the associations between each social factor and BHS change sub-scores, estimated using linear regression models stratified on sex. Models 0 was adjusted for age, baseline BHS, and time between measurements. Models 1 was additionally adjusted for previously selected confounders and social factors considered causally prior to the exposure of interest, providing an estimate of the exploratory total effect of each social factor on BHS change. Models 2 was further adjusted for previously selected mediators and social factors considered causally subsequent to the exposure of interest, providing an estimate of the exploratory direct effect.

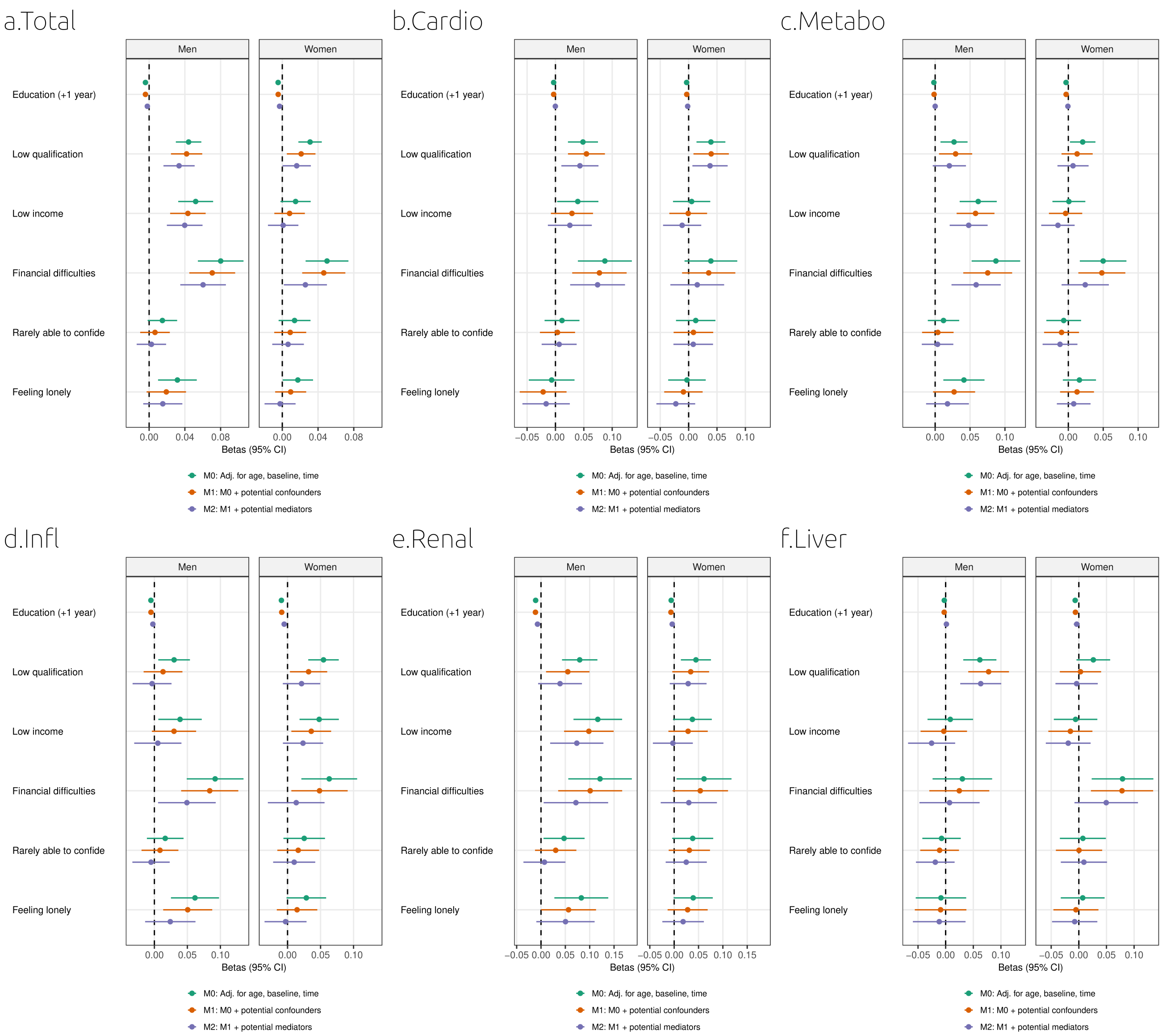

Figure S7. Sub-scores survival analysis

Hazard ratios (HRs) and 95% confidence intervals (CIs) are shown for the associations between 4-year changes in BHS sub-scores (cardiovascular, metabolic, inflammatory, renal, and liver domains) and subsequent incidence of **(a)** cancer incidence, **(b)** atherosclerotic cardiovascular disease (CVD) incidence and **(c)** all-cause mortality using age as timescale and stratified on sex. Estimates are presented for two models: Model 0 (M0), adjusted for age at baseline, baseline BHS level, and time between biomarker assessments; and Model 6 (M6), additionally adjusted for all selected potential confounders identified through the multivariate variable selection procedure performed for each sub-score and each sex. HRs represent the relative change in risk associated with a one-unit increase in the standardized change of each sub-score.

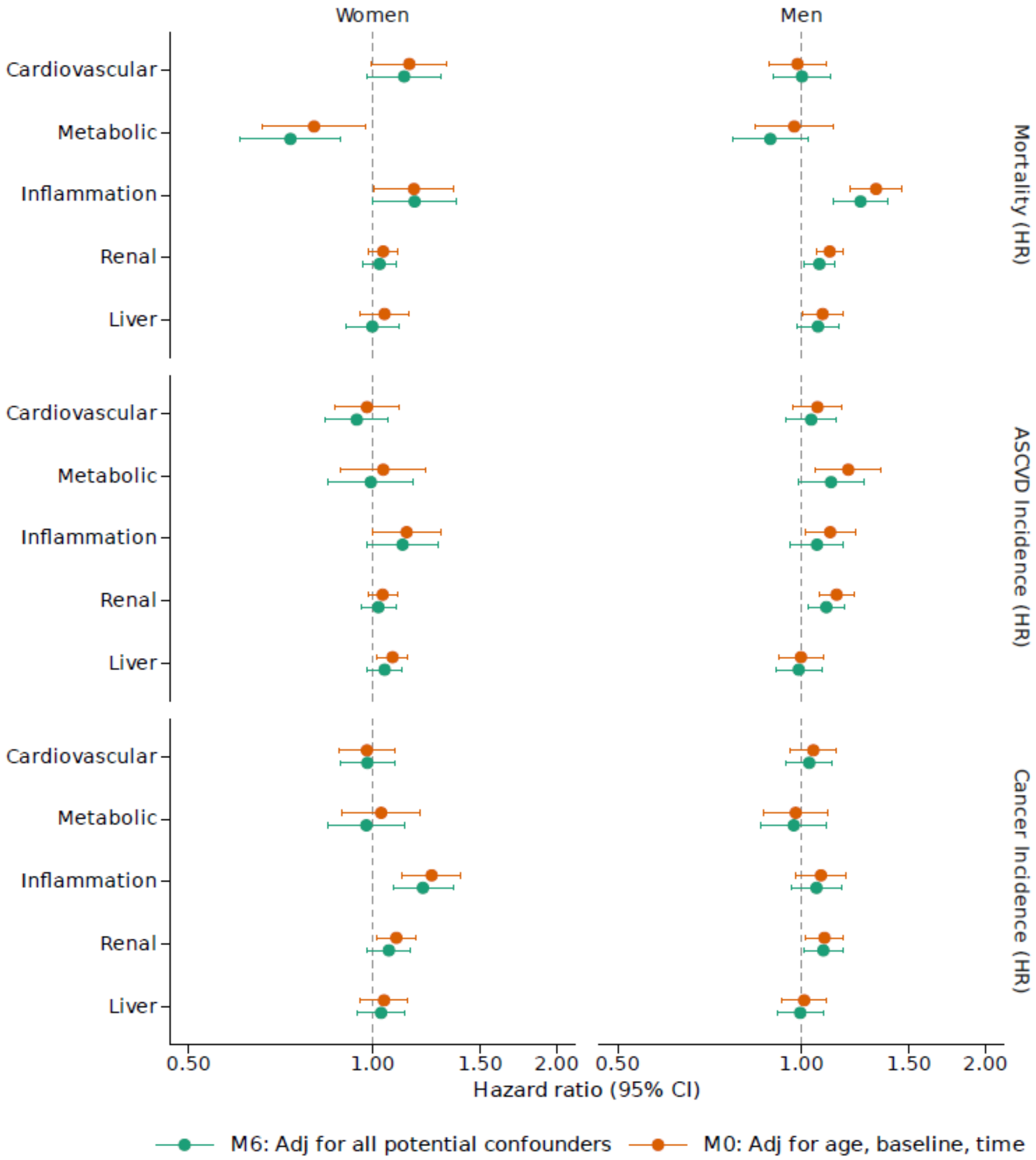

133
